# A comparative study of experimental and simulated ultrasound beam propagation through cranial bones

**DOI:** 10.1088/1361-6560/ada19d

**Published:** 2025-01-15

**Authors:** Alisa Krokhmal, Ian C Simcock, Bradley E Treeby, Eleanor Martin

**Affiliations:** 1 Department of Medical Physics and Biomedical Engineering, University College London, London WC1E 6BT, United Kingdom; 2 Great Ormond Street Hospital for Children NHS Foundation Trust, Great Ormond Street, London WC1N 3JH, United Kingdom; 3 UCL Great Ormond Street Institute of Child Health, 30 Guildford Street, London WC1N 3EH, United Kingdom; 4 NIHR Great Ormond Street Hospital Biomedical Research Centre, 30 Guildford Street, London WC1N 3EH, United Kingdom; 5 Wellcome/EPSRC Centre for Interventional and Surgical Sciences, University College London, London WC1E 6BT, United Kingdom

**Keywords:** transcranial ultrasound, focused ultrasound, k-Wave, numerical modelling, experimental validation

## Abstract

*Objective.* Transcranial ultrasound is used in a variety of treatments, including neuromodulation, opening the blood–brain barrier, and high intensity focused ultrasound therapies. To ensure safety and efficacy of these treatments, numerical simulations of the ultrasound field within the brain are used for treatment planning and evaluation. This study investigates the accuracy of numerical modelling of the propagation of focused ultrasound through cranial bones. *Approach.* Holograms of acoustic fields after propagation through four human skull specimens were measured for frequencies ranging from 270 kHz to 1 MHz, using both quasi-continuous and pulsed modes. The open-source k-Wave toolbox was employed for simulations, using an equivalent-source hologram and a uniform bowl source with parameters that best matched the measured free-field pressure distribution. *Main results.* The average absolute error in k-Wave simulations with sound speed and density derived from CT scans compared to measurements was 15% for the spatial-peak acoustic pressure amplitude, 2.7 mm for the position of the focus, and 35% for the focal volume. Optimised uniform bowl sources achieved calculation accuracy comparable to that of the hologram sources. *Significance.* This method is demonstrated as a suitable tool for prediction of focal position, size and overall distribution of transcranial ultrasound fields. The accuracy of the shape and position of the focal region demonstrate the suitability of the sound speed and density mapping used here. However, large errors in pressure amplitude and transmission loss in some individual cases show that alternative methods for mapping individual skull attenuation are needed and the possibility of considerable errors in pressure amplitude should be taken into account when planning focused ultrasound studies or interventions in the human brain, and appropriate safety margins should be used.

## 1. Introduction

As transcranial ultrasound therapies evolve, accurate treatment planning remains challenging. The aim of this paper is to evaluate the accuracy of simulations of ultrasound propagation through cranial bone, for subject-specific planning of transcranial ultrasound therapies. Despite advances in simulation methods, there are few examples of quantitative validation of models, which is crucial to ensure their accuracy and consequently the efficacy and safety of treatments.

Applications of transcranial ultrasound are diverse: focusing ultrasound through the skull is used for neuromodulation (Fomenko *et al* 2018, Kamimura *et al* 2020, Stern 2021), thermal ablation (Coluccia *et al* 2014, Franzini *et al* 2020), mechanical ablation of brain tissues using boiling (Rosnitskiy *et al* 2019) and shock wave histotripsy (Sukovich 2018), opening the blood brain barrier (Reinhard *et al* 2006) and imaging (Yasuda *et al* 2019). Despite the variety of applications, some of which are in clinical use, accurate prediction of acoustic beam propagation through the skull bones remains challenging, due to skull induced aberration and attenuation. The heterogeneous structure and shape of the skull causes significant distortion of the ultrasound field, changing the position and size of the focal region (Sigona *et al* 2023). Uncertainty in the acoustic properties of the skull contributes to uncertainty in prediction of the *in situ* pressure field (Stanziola *et al* 2023). Empirical models are often used to describe skull properties such as sound speed or attenuation coefficient (Aubry *et al* 2003, Marquet *et al* 2009, Pinton *et al* 2012), but these relationships were obtained from limited numbers of skull samples, so may not accurately represent the actual acoustic characteristics of the individual skull (Fry and Barger 1978).

The main task in planning an ultrasound intervention is the assessment of the acoustic exposure and focal characteristics. For multi-element array transducers, numerical modelling is also used to determine the phase and amplitude of the signals required to achieve focusing and aberration correction (Jones and Hynynen 2015). Calculations of heating due to absorption of ultrasound are performed to estimate the volume of treated tissue in thermal ablation therapies, (Pasquinelli *et al* 2019), and for safety evaluation in neuromodulation studies (Ding *et al* 2015). Different numerical models and methods have been used previously to simulate the propagation of ultrasound through the skull, including finite difference methods (Deffieux and Konofagou 2010), hybrid angular spectrum method (Almquist *et al* 2016, Leung *et al* 2019), finite element methods (Salahshoor *et al* 2020), k-space pseudospectral methods (Maimbourg *et al* 2018), hybrid methods combining numerical calculations and analytical models (Marsac *et al* 2017), and the ray-tracing method (Clement *et al* 2004). Each method has its own advantages and disadvantages in terms of the physics captured by the model and computational load. Modelling intercomparisons have shown that all of these methods can agree well when simulating ultrasound with the same source and medium inputs, which suggests some level of accuracy (Bancel *et al* 2021, Aubry 2022). However the final step is to perform experimental validation which incorporates other practical factors involved in using models to simulate real situations, such as determining material properties and registration. Several studies have previously experimentally verified the accuracy of simulations, but often through verification of aberration correction with multi-element transducers (Clement and Hynynen 2002, Almquist *et al* 2016). For example, simulations were used to determine the phases required to correct for aberrations induced by a plastic skull mimicking phantom and an *ex vivo* skull sample using a 256 element spherically focusing array transducer (Almquist *et al* 2016). With the phantom, when the acoustic properties were well known, simulation-based correction recovered 95% of the peak pressure achieved with reference hydrophone correction, demonstrating the accuracy of the model under these conditions. However, for the *ex-vivo* skull, simulation-based corrections recovered only 70% of the reference pressure, emphasising the impact of uncertainties in skull acoustic properties on simulation accuracy. Only a few studies have directly compared measured and simulated acoustic fields inside the skull cavity, with mapping of acoustic characteristics from CT images, albeit in a rather narrow frequency range. For example, for transmission of 60 and 120 kHz ultrasound through four *ex vivo* human skulls with incidence angle below 24^∘^, mean errors in predicted peak pressure amplitude were $\lt$14% (Bouchoux *et al* 2012). In another study, the accuracy of focal position and dimensions were within 3 mm, and spatial peak pressure was 5% when a finite difference method was used to simulate propagation of a 250 kHz field through *ex vivo* ovine skulls (Yoon *et al* 2018). Several studies have compared acoustic and thermal simulations to clinical MR thermometry (Leung *et al* 2019, McDannold *et al* 2019). While they do not make direct comparison of acoustic fields, these studies made comparisons with *in-vivo* temperature measurements from a large number of patients and sonications, showing uncertainty in focal spot temperature rise within 5 ^∘^C–10 ^∘^C.

The open-source k-Wave toolbox (Treeby and Cox 2010) and k-Plan, an advanced modeling tool with a user-friendly interface for the planning of transcranial ultrasound procedures (Brainbox Ltd Cardiff, UK), are becoming increasingly widely adopted for planning and verification in transcranial ultrasonic neuromodulation in human (Mueller *et al* 2017, Bao *et al* 2024, Martin *et al* 2024) and animal studies (Chaplin *et al* 2018). However, its accuracy in simulating intracranial ultrasound fields where the acoustic medium properties are mapped from from medical images, has not yet been fully evaluated. This paper builds on previous k-Wave validation studies. For example, it has been shown previously that after propagation through a planar or wedge-shaped glycerol-filled phantom, the difference in measured and simulation focal position was 0.7 and 1.4 mm respectively, and difference in spatial-peak pressure amplitude was 8.5% and 10.7%, respectively, which was similar to the expected uncertainty on hydrophone measurements (Martin *et al* 2019). In this study, the sound speed and attenuation coefficient of the medium were well-quantified, but errors may have been caused by uncertainties in the nonlinearity parameter and phantom geometry, and systematic uncertainty in the hydrophone sensitivity. In another study, experimental validation of linear k-Wave simulations were performed using 3D printed phantoms derived from CT scans of a human skull (Robertson *et al* 2017). Again the medium properties and geometry were well quantified, and in this case the simulated and measured spatial peak pressure amplitudes agreed to within 1%, and the focal position to within 1 mm. These validation studies demonstrate that when the medium properties are well characterised, accurate quantitative predictions of acoustic field propagation through absorbing media can be obtained using k-Wave.

However, for simulations performed for person-specific planning or evaluation of transcranial ultrasound therapies, the geometry of the skull must be obtained from CT scans, or MRI images converted to pseudoCT images. The material properties are mapped or assigned from these images, leading to potentially large uncertainties in sound speed and attenuation coefficient. As discussed in a recent review of transcranial ultrasound simulations, one of the major challenges is knowing the skull properties, which vary significantly among individuals (Angla *et al* 2023). While there are a number of studies linking sound speed to density obtained from CT scans, which have been implemented by other researchers (Douglas Mast 2000, Connor *et al* 2002, Aubry *et al* 2003, Pichardo *et al* 2010), demonstration of mapping of attenuation coefficient from image values or density is more limited, and consensus is lacking (Pichardo *et al* 2010, Leung *et al* 2019, Webb *et al* 2020). Another factor which adds to uncertainty in acoustic properties mapped from medical images is the low resolution of clinical images in comparison to the size of internal structures of the skull (Alexander *et al* 2019). This leads to spatial averaging, and incorrect description of scattering effects within the skull bone. Given these sources of uncertainty in the acoustic properties of the skull, it is important to evaluate simulation accuracy with current methods to establish the utility of simulation in transcranial therapies.

In addition to well defined medium properties, simulations also require an accurate description of the acoustic source. In order make full quantitative comparisons between measurement and simulation, a measurement based source definition is required to fully describe the source input. At the simplest level, a simplified source model can be used to replicate the measured field accurately at least over the focal region. For a spherically focusing source, this is characterised by the aperture diameter and radius of curvature, which are optimised to minimise differences between the simulated field and measured pressure along the beam axis and lateral to the focus. A uniform pressure amplitude and phase is then assigned over the entire source. This approach provides a convenient source model for planning transcranial interventions in clinical applications (Young Park *et al* 2019, Atkinson-Clement *et al* 2023), and is implemented in k-Plan. The most comprehensive source definition is based on a measured hologram (planar measurement of the complex or time varying pressure) from which the complex or time varying source surface pressure can be reconstructed (Sapozhnikov *et al* 2006). This is more complicated and time consuming to measure, and may be challenging for multi-element arrays. It requires some extra simulation steps to obtain the source definition, and higher memory requirements in the simulations.

While the simpler uniform pressure source can replicate the field very well over the focal region, the near field region can differ substantially from the measured field (Sapozhnikov *et al* 2015, Treeby *et al* 2018). In transcranial ultrasound therapies, this is where the skull is positioned, and it is not known what the affect of errors in this region will be on the simulated field either near the skull or close to the focal region. Assessment of skull heating during transcranial therapies is also important, as hot spots could lead to heating of the scalp or cortical brain tissue close to the skull, resulting in undesirable effects (Leduc *et al* 2012, Schwartz *et al* 2018). Since heating is often assessed by simulation, it is crucial to assess differences in pressure at the skull for the two sources, which may lead to differences in either the magnitude or location of predicted heating. We therefore aim to investigate the extent to which simulated fields are affected by use of a simpler source model to assess the utility of these models in planning transcranial ultrasound exposures.

In summary, this work seeks to validate the accuracy of k-Wave simulations against hydrophone measurements over a range of sub-MHz frequencies commonly used in transcranial ultrasound, for a set of *ex-vivo* human skulls. The simulations conducted in this research use the same skull mapping methods and calculation parameters as those used in k-Plan, making the findings applicable for k-Plan users planning transcranial interventions. Simulations are performed with both short pulses, to allow easier comparison of pulse arrival times and predictions of multiple reflections, and in quasi-continuous wave mode to more closely replicate conditions during the long pulses employed in ultrasonic neuromodulation. This builds on previous validation studies which show that when the medium properties and geometry are well known, simulation accuracy is high. Here we will assess the accuracy of k-Wave fluid simulations with acoustic properties and geometry of the skull mapped from CT images. We will also investigate the difference between fields simulated using uniform pressure sources and with hologram derived sources, where the skull is positioned in the near field, which is most affected by the choice of source definition.

## 2. Experimental methods

### 2.1. *Ex-vivo* human skull bones

Four human skulls were obtained under a material transfer agreement in accordance with the UK Human Tissue Act. The skulls are old and have been cleaned and stored dry. The skulls, labelled with codes A, C, B, and D, varied in porosity, surface smoothness, shape and size. They had been previously cut to separate the calvaria from the lower part of the skull. The calvaria only were used in this study. Prior to experiments and imaging, skulls were placed in a container filled with deionised water and degassed for 48 h at −400 mbar.

To facilitate mapping of the skull acoustic properties, the skulls were CT scanned using a Siemens SOMATOM Force CT scanner using a ‘Head Spiral’ protocol, with tube voltage 120 kVp, exposure time 1000 ms, x-ray tube current 137 mA, exposure 228 mAs, and flat filter. The image was reconstructed using the $Hr69h\backslash3$ convolution kernel suited to bone imaging. The in-plane pixel spacing was $0.47 \times 0.47$ mm, and the slice thickness was 0.5 mm. An electron density phantom (model 062 M, CIRS, Norfolk, VA, USA) with inserts of densities 1000–2200 kg m^−3^ was scanned with the same imaging protocol to calibrate the relationship between Hounsfield units (HUs) and density.

### 2.2. Experimental setup

Measurements and simulations were performed at frequencies between 270 kHz and 1 MHz, in both pulsed and quasi-continuous wave modes (PW and CW) with the source defined in simulation using both a hologram and a uniform pressure bowl source, in order to investigate the dependence of simulation accuracy on frequency, emission mode, and source definition.

Three spherically focusing ultrasound transducers (Sonic Concepts, Bothell, WA, USA) were used to generate acoustic fields. A 2-element (central bowl with outer ring with equal area) H115 transducer was driven at 270 kHz with a 2-channel TPO (Sonic Concepts, as above) via an electrical impedance matching network, a single element H104 transducer was driven at 500 kHz, and a single element H101 transducer was driven at 750 kHz and 1 MHz, both with an arbitrary waveform generator (33500B, Keysight, Berkshire, U.K.) and E&I 1020 L RF amplifier (Electronics and Innovation Ltd Rochester, NY, USA) via their electrical impedance matching networks. All transducers featured piezoelectric concave elements with a nominal aperture radius of 32 mm and radius of curvature of 63.2 mm.

To characterise the transmitted acoustic fields, measurements were performed using a hydrophone mounted in an automated scanning tank filled with deionised water. Across the series of measurements, three hydrophones with submersible preamplifiers were used: two 0.2 mm PVDF needle hydrophones (Precision Acoustics, Dorchester, U.K.), and a 0.2 mm capsule-type hydrophone (HGL0200, Onda, Sunnyvale, CA, USA). To minimize the impact of reflections from the hydrophone holder, the area around the hydrophone was covered with a piece of acoustic absorber. The water temperature was maintained at 20 ^∘^C, with a variance of $\pm2\,^\circ$C across experiments. Waveforms were acquired, digitized, and stored via digital phosphor oscilloscope (DPO5034B, Tektronix U.K. Ltd Berkshire, U.K.), controlled by the scanning tank software, with a sample rate of 125 MHz and 32 averages.

### 2.3. Source characterisation and free field validation measurements

Each transducer was characterised to obtain source definitions for use in simulations. Transducers were characterised in 2 ways: as a planar equivalent mass source calculated from a measured hologram, and as an equivalent bowl source with uniform distribution of pressure on a spherical cap surface.

Holograms were measured at all frequencies in both CW and PW modes under free field conditions. In PW, the transducer was driven with a 2-cycle sinusoidal pulse at the chosen frequency. In CW, the signal was a 60 cycle sinusoidal pulse. The pulse repetition interval was 10 ms in both cases. For the 270 kHz case, the TPO was driven with PW and CW signals consisting of 10 *µ*s and 60 *µ*s pulses respectively, with a 10 ms repetition interval. The peak-to-peak drive voltage applied to the matching network ranged from 12 to 33 V, across transducers, adjusted according to the sensitivity of the hydrophone used for the measurement. The TPO power was set to 4 W.

Short pulses were used to directly observe the propagation and reflection of wave packets, analyse the arrival time of signals, and minimise the influence of reflections between the skull, transducer, and hydrophone. At the same time, CW signals are of great interest, as they more realistically represent therapeutic protocols. For PW measurements, the acquisition window included the entire wave packet covering the earliest and latest arrival times of the pulse at the measurement positions. In CW mode, a section including 10–20 wave periods with stable amplitude was recorded. For both PW and CW modes, the acquisition window was short enough to exclude the signal reflected from the hydrophone.

For source characterisation, the beam axis of the transducer was aligned with the z axis of the scanning tank by finding the location of peak pressure at two axial positions. The angular alignment of the transducer was adjusted according to the angular offset between the beam axis and scanning tank axis. The final discrepancy between axes was ${\lt}0.1^\circ$. The holograms were captured in a plane perpendicular to the beam axis, between the transducer and the focal region, at axial distances between 40–45 mm for the different sources. The scan plane size ranged from 60 mm at 1 MHz to 100 mm at 270 kHz, with a spatial step size of 0.5 mm.

To construct the source holograms, the equivalent mass source on a plane coincident with the physical source origin was calculated using the equivalent source method with the calculateMassSource function in k-Wave (Treeby *et al* 2018). This source, when used as input to k-Wave, reconstructs the measured data. The signals at each point were first band pass filtered (0.1 MHz–3 MHz). CW signals were cropped to an integer number of cycles, and PW signals were windowed using a Tukey window, before the spectrum was obtained by FFT. The frequency dependent hydrophone sensitivity was applied to obtain acoustic pressure from the voltage signals.

In addition, for each transducer and frequency, equivalent uniform amplitude bowl source parameters were determined (table 1). The nominal specifications of a focusing transducer do not always match the effective values, which best describe the emitted field. To obtain these parameters, the axial pressure distributions were measured in CW mode. The axial pressure amplitude can be described analytically by O’Neil’s solution for a spherically focusing transducer driven by a continuous sinusoid at a given frequency with uniform normal surface velocity (O’Neil 1949). Using gradient descent optimisation, the radius of curvature *r_o_* and aperture radius *a_o_* of the emitting bowl were obtained by minimising the difference between the analytical and measured axial pressure.

**Table 1.** Optimised parameters of equivalent bowl sources to match the transducers which have nominal radius of curvature 63.2 mm and aperture radius 32 mm.

Transducer	*f* (kHz)	*r_o_* (mm)	*a_o_* (mm)
H115	270	61.39	25.20
H104	500	60.07	28.19
H101	750	63.41	29.95
H101	1000	62.89	30.63

### 2.4. Skull positioning and validation measurements

**Figure 1.**
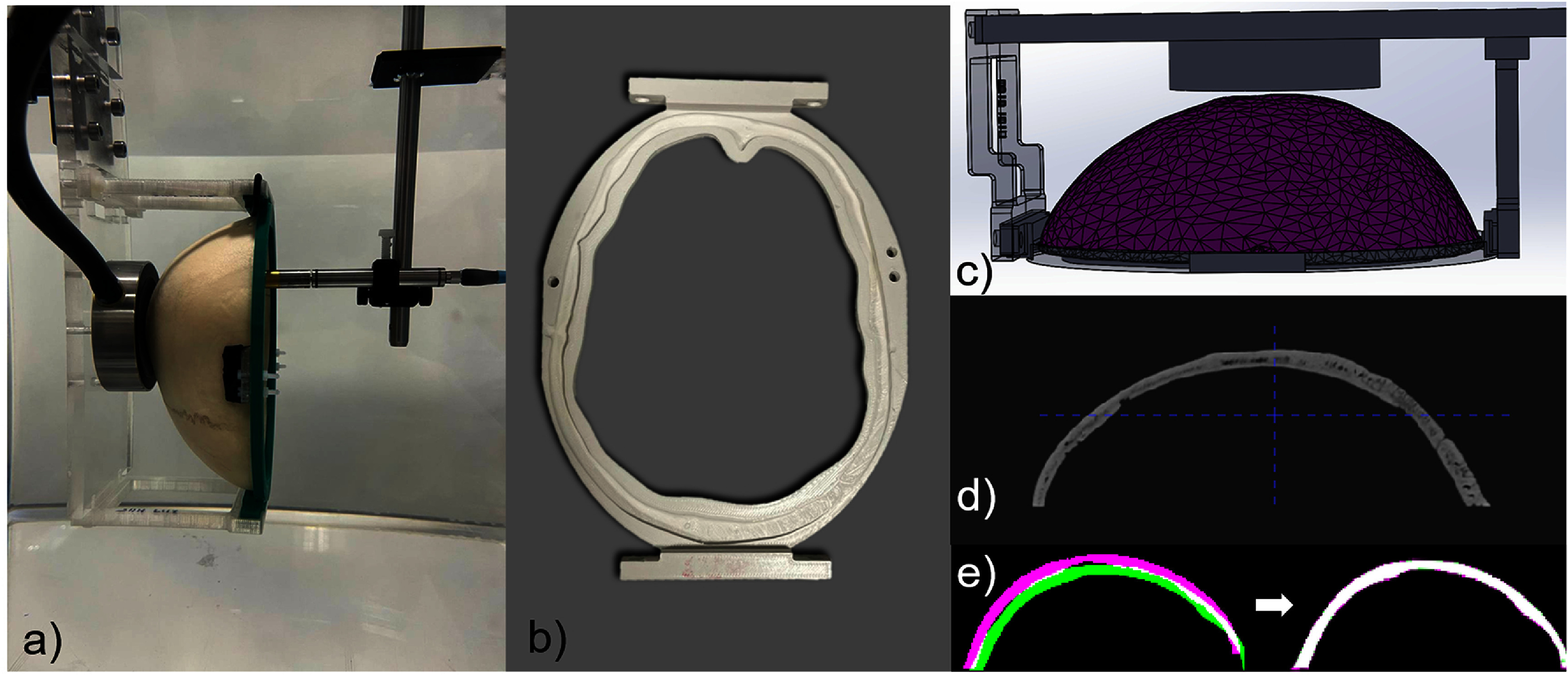
(a) Experimental setup showing hydrophone scanning within the cavity of an *ex-vivo* skull calvarium which is held in position by a (b) 3D-printed plastic skull holder and mounts attached to the transducer mounting plate; (c) 3D CAD model of the experimental setup; (d) CT image of a skull; (e) CT image mask and skull mesh registration.

For precise transducer and skull positioning, 3D CAD models of both were created (figure 1(c)). The transducer model was defined geometrically using specifications obtained from the manufacturer. Skull models were created from a mesh derived from CT scans. The CT images were thresholded using 3D Slicer (Fedorov 2012) (www.slicer.org/), then morphological closure was performed to fill small holes before Gaussian smoothing was applied. The resulting segmentation was exported as a.stl file. Meshmixer (Autodesk Research, London, UK) was then used to repair, optimise, and reduce the mesh. The transducer and skull meshes were positioned together in Solidworks (Dassault Systèmes SolidWorks Corporation, Waltham, MA, USA) with the highest point of the skull located 10 mm from the central point of the transducer surface, with the base of the skull section parallel to the back of the transducer housing.

Skulls were precisely positioned in holders which had a notch replicating the shape of the skull base created by subtracting the skull mesh from the holder body (figure 1(b)). Skulls were held in place using clamps screwed into the holder. Custom connecting parts were then designed within the SolidWorks assembly to rigidly mount each skull on the transducer mounting plate in the correct position, as illustrated in figure 1(c). The transducer was mounted using screws on this plate which was fabricated from laser cut perspex. All other mounts and holders were 3D printed in PLA using an UltiMaker S5 (Ultimaker B.V, Geldermalsen, NL). Due to deformation of the plastic and swelling of the skull during soaking, the precision of the skull position was estimated to be within ±1 mm. The transducer-skull assembly was mounted in the scanning tank using optical posts and an optical breadboard.

Before skull measurements, the transducer was mounted and the hydrophone was aligned to the beam axis in the focus. The axial position was then determined from the arrival time of the signal. Line scans of the axial pressure profile were measured for each case immediately before the skull was mounted. This was used to normalise the source amplitude used in each simulation.

In this configuration where the transducer was geometrically fixed to enable skull mounting, it was not possible to precisely adjust the angle between the beam axis and the scanning tank *z*-axis. Therefore, in free field, the angular offset was determined as described above. A correction was made to the measured hologram to align the hologram plane perpendicular to the scanning tank’s *z*-axis using a simplified correction algorithm, as described in Kaloev *et al* (2022).

Subsequently, the skull was mounted in the holder, and a hologram was obtain by hydrophone measurement on a plane perpendicular to the beam axis after propagation through the skull, 55 mm away from the transducer, as shown in figure 1(a). The scanning area was typically 60 × 60 mm, with a step size of 0.5 mm. The centre of the scan area was aligned with the position of the beam axis in the absence of the skull. Measurements were performed at all four frequencies in both PW and CW modes. The pressure was then obtained from the measured voltage waveforms as described in section 2.3. The angular spectrum method was used to reconstruct the acoustic pressure field starting at the skull surface and reaching an axial distance of 90 mm. The peak positive pressure was determined at each point, and compared with the simulated field.

## 3. Simulation methods

### 3.1. Sources

Two sets of simulations were conducted using different source definitions: one based on the equivalent mass source hologram, and the other using a bowl source with a uniform amplitude with effective curvature radius *r_o_* and aperture radius *a_o_*.

The k-Wave functions calculateMassSource and calculateMassSourceCW were used to calculate the source from the measured holograms. The emitted signal duration was sufficient to allow for reflections within the skull. For PW mode the entire signal was used to calculate the mass source, while for CW mode the amplitude and phase of the spectrum at the specified frequency were used.

The bowl source was created using the makeBowl function in k-Wave, specified by the source coordinates, aperture diameter, and radius of curvature. The bowl source represents the entire concave surface vibrating in phase. Consequently, for the PW mode, a signal derived from a point near the centre of the measured hologram was applied across the entire source surface. The amplitude of this equivalent source was scaled to match the measurements, as described in section 3.4.

### 3.2. Simulation parameters and hardware

The open-source k-Wave toolbox (version 1.4) was used to simulate ultrasound propagation through the cranial bone. Table 2 displays the simulation parameters, including temporal step size, Courant–Friedrichs–Lewy (CFL) number, number of time steps, and memory usage. The grid size was large enough to cover the extent of focal region and the plane containing the source, with a spatial step size chosen to give a minimum of 8 points per wavelength (Robertson *et al* 2017). The computational domain boundaries were surrounded by a 20-point-thick perfectly matched layer. Most simulations were performed on a high-performance computing server using parallel computations on GPUs (NVIDIA Tesla P40, 24 GB, 346 GB/s) and took up to 3 h depending on the domain size. The largest simulations, which did not fit into the GPU memory, were performed using a CPU (Intel(R) Xeon(R) CPU E5-2620 v4 @ 2.10GHz), taking up to 34 h.

**Table 2.** Simulation parameters for the largest simulation domain used to accommodate skull specimen D. Timestep, CFL and domain size varied slightly to accommodate each skull, and for the different source conditions.

*f* (kHz)	$\textrm{d}x$	Grid size (*x, y, z*)	$\textrm{d}t$ (ns)	CFL	Memory (MB)	No. timesteps	$T_\textrm{simulation}$	Device
270	0.35	344 × 394 × 394	11.7	0.06	15 646	17 078	40 min	GPU
500	0.3	386 × 384 × 384	11.7	0.1	8712	9092	25 min	GPU
750	0.2	578 × 576 × 576	7.9	0.1	23 407	13 379	3 / 34 h	GPU / CPU
1000	0.15	254 × 670 × 670	6.3	0.1	21 822	10 978	130 min	GPU

For calculations at 1 MHz, the size of the numerical model, extending 90 mm from the source, was too large to fit into GPU memory, and CPU calculations would have taken several days. In this case, the size of the computational domain was reduced to 35 mm axially, and the transient pressure field was calculated at each point on a plane 30 mm from the transducer. The angular spectrum method was then used to propagate the field over the volume of interest.

### 3.3. Medium geometry and properties

To calculate the position and orientation of the skulls relative to the transducers, the CT images of the skulls were registered with the skull meshes from the skull-transducer CAD assemblies, as shown in figure 1(e). Initially, the CT images in.nii format (figure 1(d)) were imported to MATLAB, and coarsely aligned with the skull mesh. The CT HUs from the CT images were transformed into density maps using the CT calibration curve, then a skull mask was generated by thresholding the density map at 1150 kg m^−3^. All small sub-threshold inclusions within the main volume (holes) were filled, and small unconnected islands outside the main volume were discarded, retaining only the largest connected component in the mask. The skull meshes were converted to binary masks with skull-transducer separation determined by the CAD assembly, oriented such that the *z* = 0 coordinate corresponded to the source plane, with the *XY* plane centered on the source.

The CT image mask and skull mesh were initially resampled onto a 1 mm isotropic grid for coarse and rapid registration (figure 1(e)) using the MATLAB imregtform function, which employs a gradient descent method to determine translational and rotational matrices. The registration was repeated after resampling onto a grid with the same resolution as the computational grid to refine the registration. The original CT image was then resampled onto the computational grid by applying the resulting transform using linear interpolation. The region surrounding the skull was assigned the properties of water at a temperature of 20 ^∘^C.

Cranial bone density *ρ* was calculated from CT HUs Sound speed *c* was linearly mapped from density as Marquet *et al* (2009)

\begin{equation*} c = \rho \cdot 1.333 + 166.7.\end{equation*}

**Figure 2.**
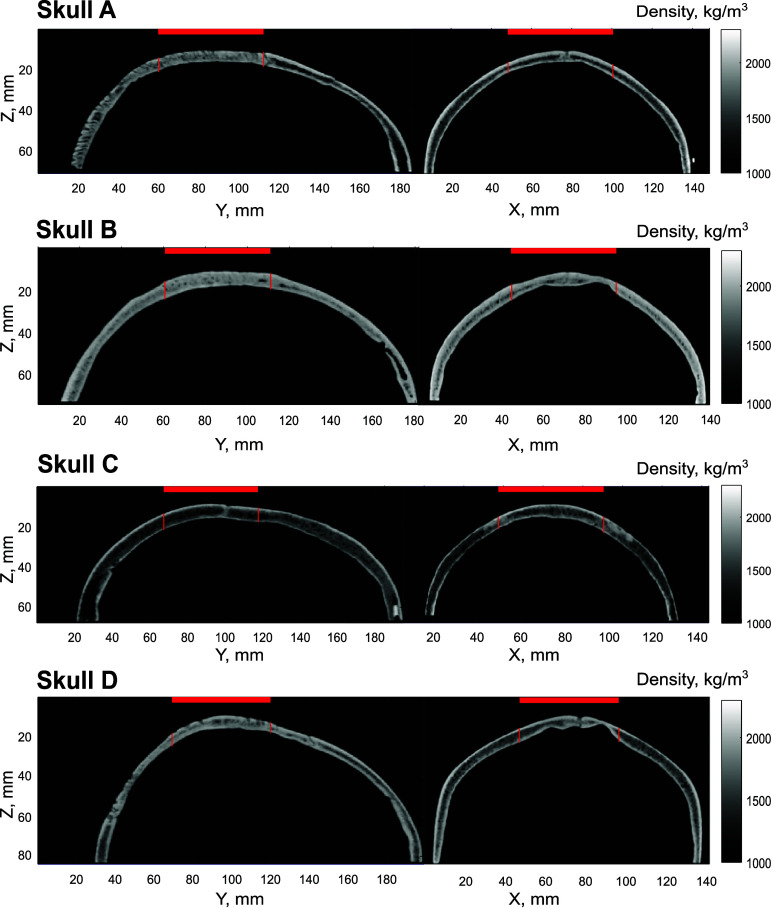
Density maps of 4 skull specimens, obtained from CT scans. The horizontal red lines show the position and extent of the transducer, vertical lines mark the corresponding section of the skull over which average density was calculated..

In figure 2, cross-sectional density maps of the four skull are shown. Variations in skull characteristics can be seen: for instance, skull C is thicker and more porous, while skull B is more dense. The skulls differ also in shape: skull D is the largest, while skull C is the smallest; skulls B and D have a very heterogeneous shape in the central area, with large variations in thickness, while skulls A and C are quite uniform in their thickness. Skulls A and D are marked by irregularities, depressions, and fissures. The average thickness *D* in the central 50 mm region of the skulls obtained from the images was between 6.1 and 7.5 mm. The average sound speed, density and thickness of each skull over this region are presented in table 3.

**Table 3.** Average ± standard deviation of speed of sound *c*, density *ρ*, and thickness *D* of the skull specimens over the 50 mm central region.

No.	*c* (m s^−1^)	*ρ* (kg m^−3^)	*D* (mm)
A	2403$\,\pm\,$341	1677 ± 256	6.2 ± 0.5
B	2514$\,\pm\,$255	1761 ± 191	6.1 ± 1.2
C	2172$\,\pm\,$305	1504 ± 229	7.5 ± 0.7
D	2378$\,\pm\,$278	1659 ± 208	6.2 ± 1.3

Attenuation in the skull bone comprises a significant contribution from scattering from the internal microstructure, in addition to absorption within the bone itself. k-Wave models only power law absorption (conversion of energy to heat) with contributions to scattering arising from the defined heterogeneous medium. This means that scattering from the sub-grid resolution microstructure of the bone is not explicitly modelled. We therefore use the k-Wave absorption to include the bulk contribution of scattering to the attenuation. A constant absorption coefficient of $\alpha = 13.3 \cdot f$ (dB cm^−1^), taken from Pinton *et al* (2012) was applied to all voxels in the skull mask. The absorption power law exponent was set to 2 for the whole simulation domain, including the skull, to avoid numerical dispersion of the computational scheme. To define the linear power law absorption over the skull mask, the reference absorption coefficient value for skull was then modified to generate the correct absorption coefficient at the frequency of interest given the reference power law exponent value of 2.

### 3.4. Simulations and comparison metrics

Simulations were first performed for each source in free field, to verify that they accurately predicted the measured fields. The calculated axial pressure distribution was compared to the measured axial pressure acquired just before the skull was mounted, then the ratio of spatial peak pressure amplitudes $p^\textrm{exp}_\textrm{water}/p^\textrm{sim}_\textrm{water}$ was used to adjust the source amplitude for skull simulations. This normalisation was employed to minimise the error arising from uncertainty in hydrophone sensitivity and source output.

The simulated peak positive pressure was recorded at each grid point and compared with the corresponding measured values. The simulated fields were downsampled to a resolution of 0.5 mm for comparison to measurement data. Peak positive pressure and pressure amplitude in CW mode were considered equivalent as the acoustic fields were linear. In the case of simulations at 1 MHz, the angular spectrum approach was used to expand the signal over the entire volume, and the peak positive pressure was extracted.

In addition to peak positive pressure, for frequencies 270–750 kHz, the waveforms were recorded on a plane at 55 mm from the source plane, corresponding to the measurement plane in the experiment. For the 1 MHz simulations, waveforms were recorded on a plane 30 mm from the source.

For comparison of results, spatial peak pressure, location of the spatial peak pressure, and size of the −6 dB focal zone were extracted. In addition, comparison of the acoustic pressure distribution across the entire intracranial field was performed to examine the reproducibility of additional peaks and any differences in the near and far fields. The following quantities and metrics were therefore calculated:

• Spatial peak positive pressure in free field, $p_\textrm{water}$.
• Spatial peak positive pressure in the skull, $p_\textrm{skull}$.
• Spatial position of the peak positive pressure in the skull, $(x_0, y_0, z_0)$.
• Transmission loss TL [dB], the ratio of peak pressure in focus within the skull to that in the free field, TL$ = 20\cdot \log(p^\textrm{exp}_\textrm{skull} / p^{exp}_\textrm{water})$.
• −3 dB focal dimensions, $(\triangle Lx, \triangle Ly, \triangle Lz)$
• The volume of the focal −6 dB zone, *V*.
• Difference in spatial peak positive pressure, $\varepsilon_p = 100\cdot(p^\textrm{sim} - p^\textrm{exp}) / p^\textrm{exp} ~[\%]$.
• Difference in focal position, $(\triangle x, \triangle y, \triangle z) = (x_0^\textrm{sim} - x_0^\textrm{exp}, y_0^\textrm{sim} - y_0^\textrm{exp}, z_0^\textrm{sim} - z_0^\textrm{exp})$.
• Difference in −3 dB focal dimensions, $(\triangle Lx, \triangle Ly, \triangle Lz) = (L_{xyz}^\textrm{sim} - L_{xyz}^\textrm{meas})$
• Difference between simulated and measured focal volume, $\varepsilon_V = 100\cdot(V_\textrm{sim} - V_\textrm{exp})/V_\textrm{exp} ~[\%]$.
• Mean absolute errors $ < \varepsilon_p > $, $ < \varepsilon_V > $, $ < \triangle z > $, $ < \triangle L_x > $, $ < \triangle L_y > $, $ < \triangle L_z > $ for all skulls and frequencies.
• Median of samples *x*, $q_{0.5}(x)$, where *x* is *ε*_*p*_, *ε*_*V*_ or $\triangle z$ at chosen frequency or skull.

## 4. Results

### 4.1. Free field source verification

An axial scan in free field performed directly before measuring the field inside the skull was compared to simulations for each emission mode and signal amplitude used in the skull measurements.

**Figure 3.**
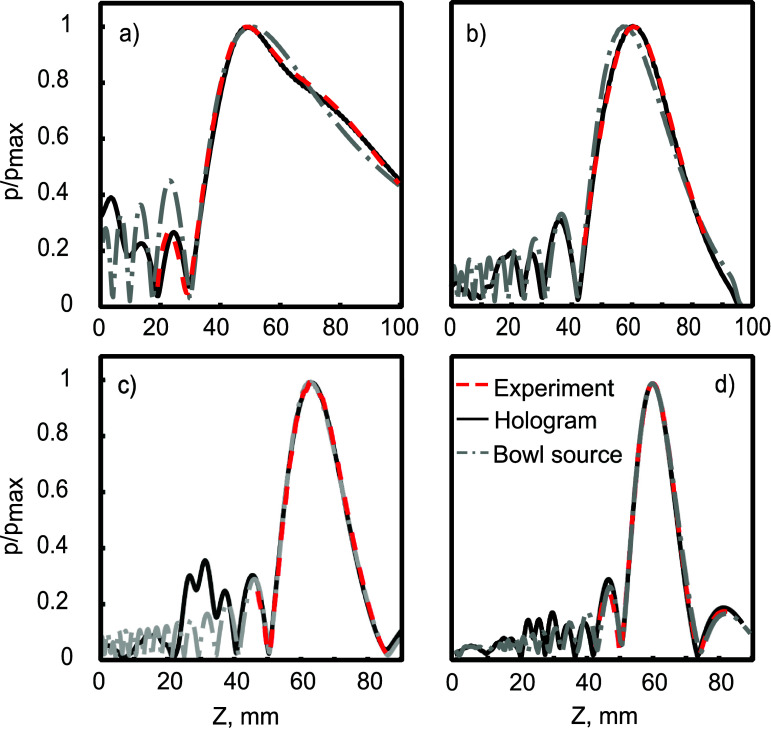
Comparison of pressure amplitude on axis in free field between experiment, k-Wave simulations using a hologram derived source, and O’Neil solution for an optimised bowl source in CW mode at (a) 270 kHz, (b) 500 kHz, (c) 750 kHz and (d) 1 MHz..

In general, the equivalent source derived from the hologram, normalised by spatial peak pressure amplitude, accurately predicts the measured axial pressure, with differences in pressure amplitude of less than 1% over the focal area, in correspondence with the results shown in Treeby *et al* (2018). Peak positions and pressure profile shapes matched well for all the frequencies investigated as shown in figure 3. The axial pressure calculated using the O’Neil solution for the optimised uniform bowl sources is also shown. It can be seen that this accurately describes the focal zone, showing an increasing divergence as it approaches the near field. In some cases, it is possible to optimise the source dimensions to obtain an accurate description of the focus and several prefocal lobes, as in the case of the 750 kHz and 1 MHz frequencies for the H101 transducer (figures 3(c) and (d)), while for the 500 kHz field with the H104 transducer, the best optimisation still results in a shift in peak pressure position of 2.5 mm ^6^6It was discovered later that an improved fit could be obtained by expanding the range of parameters included in the search. While this gave much better agreement in the focal region, the fit parameters were noticeable larger than the nominal parameters. Simulations were not repeated with these parameters. (figure 3(b)). As seen in table 1, even for the same transducer, effective source dimensions may vary slightly at different frequencies.

### 4.2. Transcranial fields

Holograms of the acoustic pressure field after propagation through each skull were measured at four frequencies in both PW and CW modes, the measurement data is available for download (Krokhmal *et al* 2024). Typically, the skull modifies the sound beam in several ways: it alters the beam shape, making it more distorted, shifts the focus, and reduces the amplitude due to additional transmission losses in the skull. The exact nature and extent of these changes depends on the shape and characteristics of the individual skull. The intracranial fields show varying degrees of aberration and absorption, which was greatest for skulls B and D.

**Figure 4.**
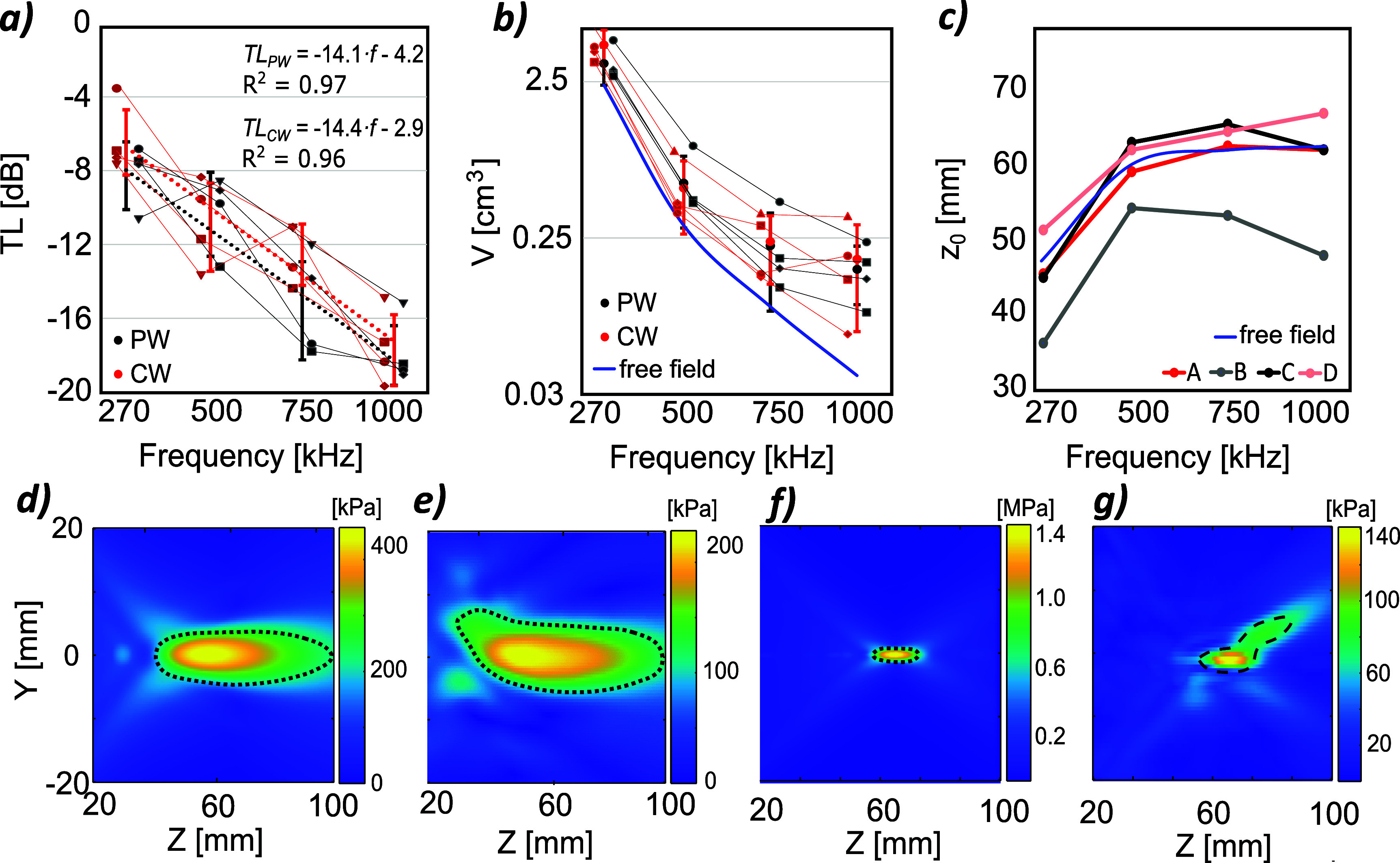
(a) Experimentally calculated transmission loss on frequency for CW (red) and PW (black) modes. Markers show results for individual skulls, the trend is indicated by dotted lines; (b) dependence of focal volume on frequency for PW and CW mode; (c) dependence of the experimental spatial pressure peak *z*-coordinate on different frequencies across skulls and in free field (blue line); peak positive pressure distribution in *YZ*-plane in free field (d), (f) and in skull D (e), (g) at 270 kHz (d), (e) and 1 MHz (d), (g). Dotted lines show boundaries of -6 dB focal zone.

As seen in figure 4(a), the average transmission loss over all samples can be described by a linear relationship with frequency. This differs slightly between pulsed and quasi-continuous emission modes due to reflections between the skull surface and the transducer: reflected waves in the pulsed mode separate into individual pulses, whereas in the quasi-continuous mode, direct and reflected waves interfere. The average transmission loss TL for PW and CW modes can be described by:

\begin{equation*} \textrm{TL}_\textrm{PW} = -14.1 \cdot f\left[\textrm{MHz}\right] - 4.2 \left[\textrm{dB}\right] ,\end{equation*}

\begin{equation*} \textrm{TL}_\textrm{CW} = -14.4 \cdot f\left[\textrm{MHz}\right] - 2.9 \left[\textrm{dB}\right] .\end{equation*}

It should be noted that the dependence of transmission loss on frequency for individual skulls is not linear, however the overall trend may be useful in estimating in general the effect of the skull.

The focal zone volume *V* is defined as the area where the acoustic pressure amplitude is no more than 6 dB below the spatial peak value. The sources used in this study have the same nominal aperture diameter and radius of curvature, so the focal size should scale with wavelength in free field under ideal conditions. As illustrated in figure 4(b), the average focal volume within the skull decreases with increasing frequency, from 4.3 ± 1.1 cm^3^ at 270 kHz to 0.18 ± 0.12 cm^3^ at 1 MHz in quasi-continuous mode as expected. However, these volumes are greater than the corresponding free field focal volumes.

At lower frequencies, regardless of skull characteristics, the acoustic beam remains well focused, with a well-defined ellipsoidal focus, as shown in figures 4(d) and (e). As frequency increases, particularly in cases where skull thickness is highly heterogeneous, the beam becomes increasingly distorted (figures 4(f) and (g)), leading to a greater discrepancy in *V* between the free field and the intracranial field. The shape of the −6 dB zone becomes complex, consisting of separate regions, making it challenging to accurately determine its volume and significantly increasing the differences between measured and simulated focal volumes. The greatest distortion was observed with skull B. The beam also experienced substantial aberration when passing through skull D, while skulls C and A caused little change in the beam structure. This appears to be correlated with the degree of skull heterogeneity: the visible distortion of the spatial peak pressure field increases with the standard deviation of the skull thickness given in table 3.

For focused transducers with the same geometry, focal position depends on frequency in free field, the focal positions are at slightly different distances from the transducer at each frequency and in particular are much closer at 270 kHz (figure 4(c)). The shift of the focal position in the skull from the free field position varies between skulls, likely depending on the shape of the skull and the resulting lensing effect. In addition, the greatest shift in the focus was observed for skull B, which also exhibited the most significant distortion. The focus remained in a more similar position to the free field location for skulls C and A which show little distortion.

### 4.3. Skull model validation

**Figure 5.**
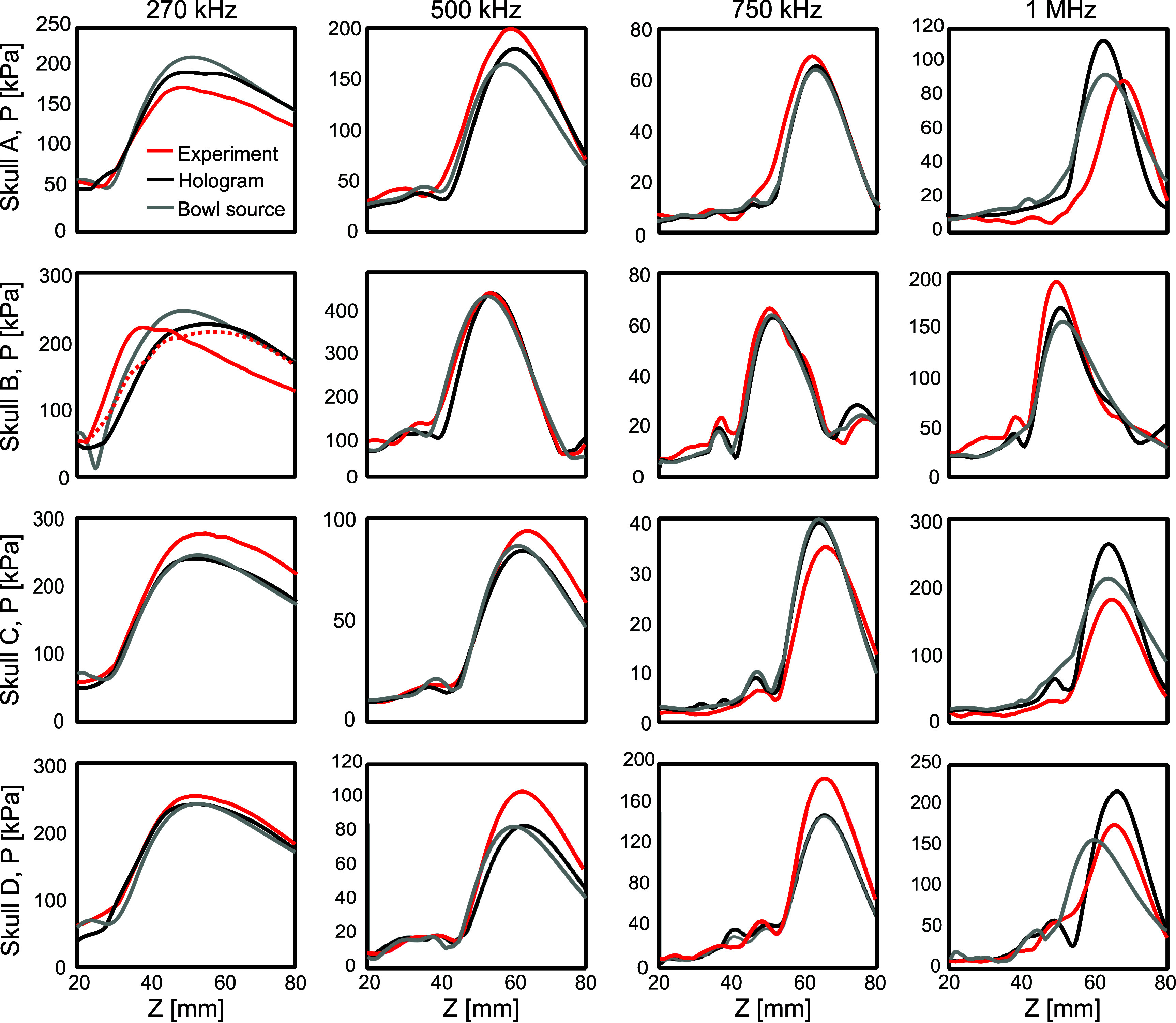
Comparison of pressure profiles passing through the spatial pressure peak within the intracranial space between experimental measurements (red) and simulations using hologram (black) and bowl sources (grey) in PW mode. For the most aberrating skull B at 270 kHz, the dotted line indicates the measured pressure profile traversing the simulation spatial peak pressure location.

**Figure 6.**
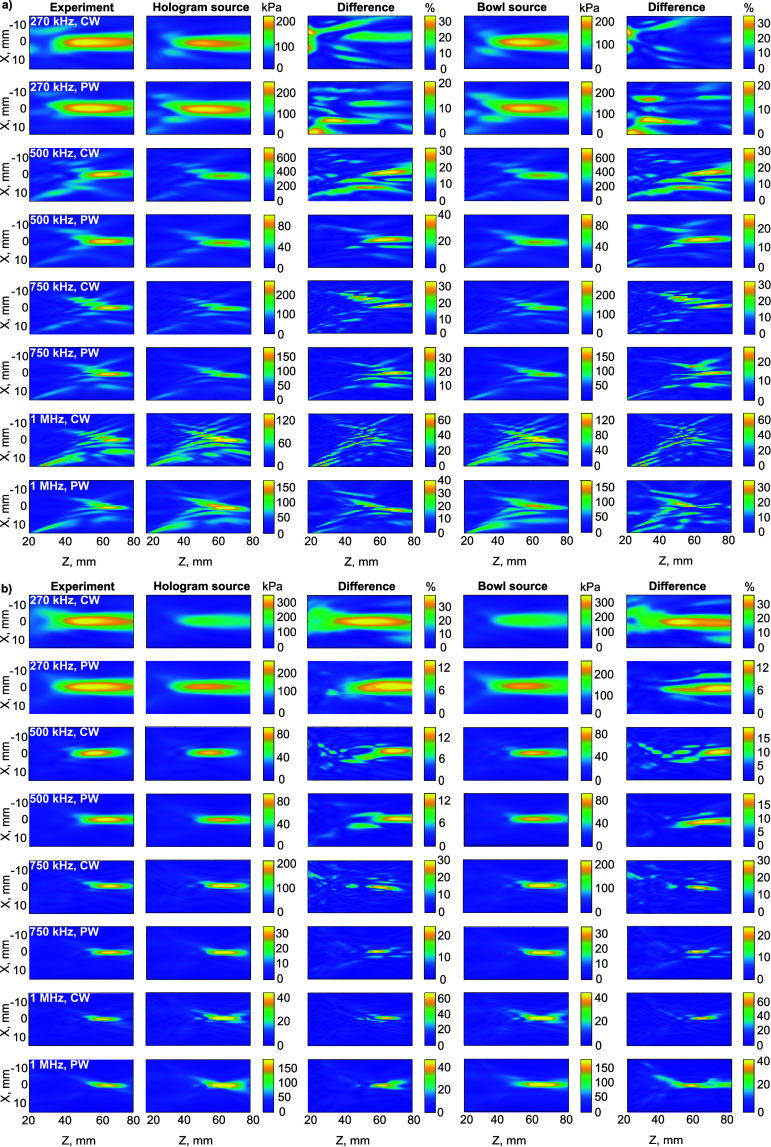
Spatial distribution of peak pressure amplitude in experiments and simulations and their difference at different frequencies, emission modes and source types for skulls (a) D and (b) C.

In general, as evident from the spatial distribution of pressure amplitude in figures 5 and 6, numerical modelling enables prediction of the beam structure, capturing the position of the focal region and additional maxima, with differences of around 15% on average in pressure amplitude. It should be noted that a good match is observed between the experimental and calculated acoustic pressure distributions both in the case of a considerably distorted field (applicable for skulls B and D, figure 6(a)) and when the skull does not significantly alter the focal structure of the beam (e.g. skulls A and C, figure 6 (b)). Along the axis of beam propagation (*Z*-axis), the maximum may be shifted, as shown in figure 5, while the lateral position of the focus is generally accurate to within the measurement step. Error in lateral focal dimensions is in general less than 1 mm, while there are larger errors in axial focal size of a few mm, up to 10 mm or more in some cases.

**Figure 7.**
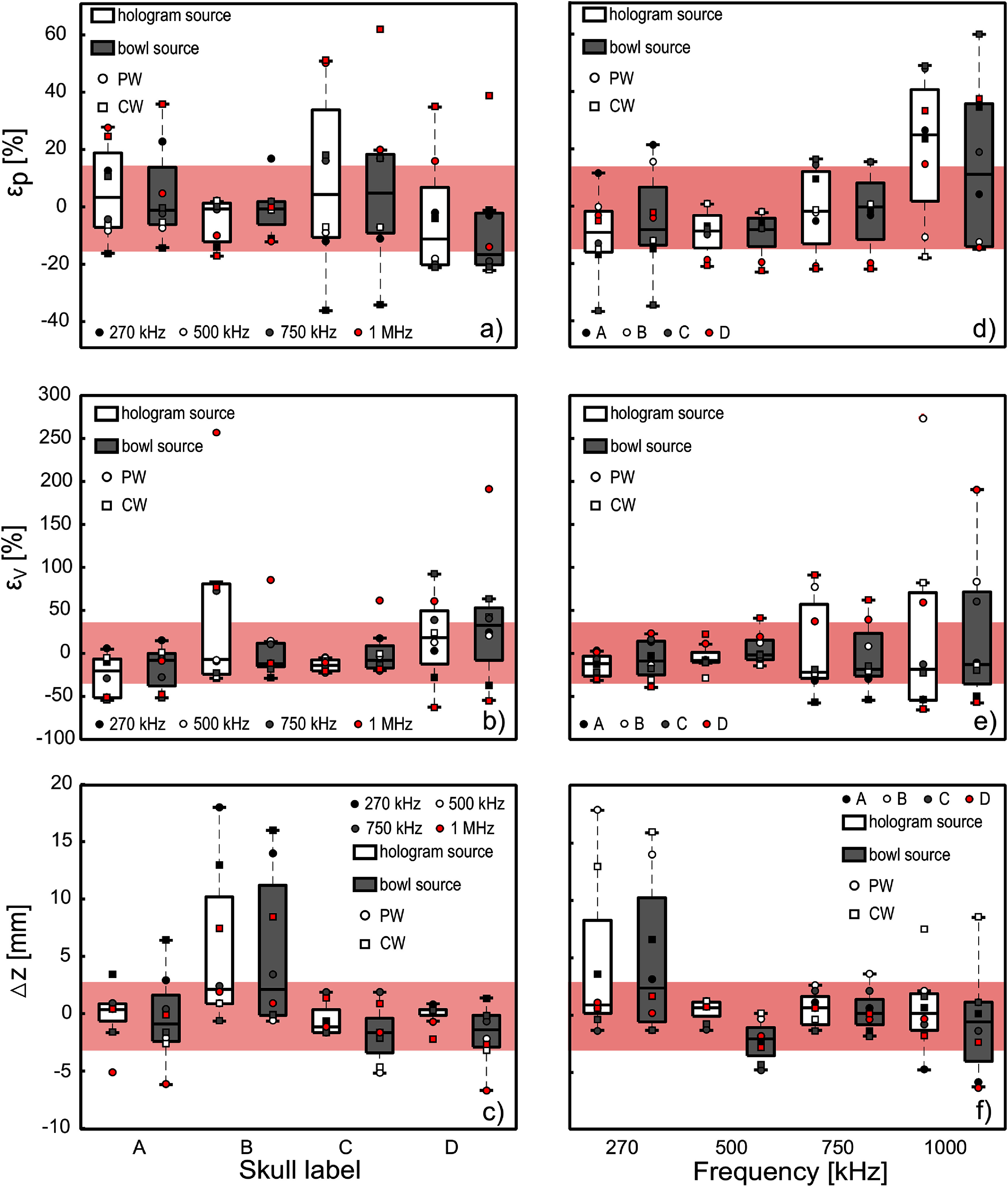
Errors *ε*_*p*_, *ε*_*V*_ and $ \triangle z$ for (a)–(c) individual skulls and (d)–(f) for different frequencies. Dots represent individual cases. Pink areas represent one standard deviation of the mean across all errors. Plotted boxes show median, upper and lower quartiles, whiskers show the non-outlier maximum and minimum values.

Figure 7 presents errors *ε*_*p*_, *ε*_*V*_ and $ \triangle z$ over all investigated cases by skull (a)–(c) and by frequency (d)–(f), grouped by source type and emission mode. Across all cases, the mean of the absolute errors and their standard deviations were $ < \varepsilon_p > = 15 \pm 13\%$, $ < \varepsilon_V > = 35 \pm 42\%$, $ < \triangle z > = 2.7 \pm 3.8$ mm, and $ < \triangle Lx > = 0.3 \pm 0.4 $ mm, $ < \triangle Ly > = 0.3 \pm 0.3$ mm, $ < \triangle Lz > = 2.3 \pm 2.3$ mm. The relatively large average $ < \triangle z > $ is attributed to the large focal position errors for skull B at 270 kHz ($\triangle z$ = 14, 18 mm). In general this skull caused significant aberration of the field, and the pressure maximum was shifted towards the transducer (shown in figure 5), although the overall position of the focal region remained similar. With this case excluded, the mean of the absolute focal shift is $ < \triangle z > = 1.9 \pm 1.9$ mm. The quantities and metrics outlined in section 3.4 are presented in tables S1–S5 in supplementary material. In general, individual errors increased with frequency, and were greater for CW mode. Errors in focal position and volume were greater for the most aberrating skull B, and errors in peak pressure amplitude were greater for the most porous skull C. Errors in focal dimensions were in general greatest at 270 kHz where the focus is longest, but there were also larger errors for the most aberrated fields at 1 MHz.

For skull B the simulated field matched the measurement extremely closely in terms of the pressure amplitude (table S2): for the first three frequencies, the error in spatial peak pressure in PW mode for the hologram source did not exceed $\varepsilon_p = 2\%$, and for measurements at 1 MHz, it did not exceed $\varepsilon_p = 11\%$. The discrepancy increased by a few % when the bowl source was used, and was greater for CW mode, with the greatest error *ε*_*p*_ observed in the CW mode at 270 kHz, reaching up to 15%. The largest discrepancies in pressure between the experimental results and numerical calculations were observed for skull C (table S3). At frequencies of 270 and 500 kHz, *ε*_*p*_ was less than 13% for all conditions except for CW mode at 270 kHz, where it reached 37%. The error *ε*_*p*_ increased further with higher frequencies, up to 50% for the hologram source and 61% for the bowl source at 1 MHz. The largest errors were seen in the spatial peak pressure at 1 MHz: the error *ε*_*p*_ ranged from 11% for the PW case with a hologram source in skull B, to 60% in the CW mode with a bowl source for skull C. For skull B, the predicted amplitude was underestimated, while for other skulls it was overestimated compared to experiment. However, at lower frequencies, the pressure amplitude tended to be underestimated for most skulls. Figure 7(b) shows the error in the simulated focal volume *ε*_*V*_ for each skull. The mean and range of errors appear similar for both sources in general, and the mean error was similar for all skull samples. However, *ε*_*V*_ varied more at higher frequency and for skull B and D which caused greater distortion of the field. Skull B also has some of the highest errors in focal dimensions in general, particularly at 270 kHz where the error is up to 40% and 50% of the axial and lateral focal length respectively. Agreement between the simulated and measured location of spatial peak pressure was generally good, as shown in figure 7(c). For most cases, the axial error $ \triangle z$ was less than 2 mm and the lateral errors $ \triangle x, \triangle y$ were less than 1 mm. Among all 64 cases considered, the error $ \triangle z$ exceeded ±2 mm in 16 of them. However, even in cases where the discrepancy in focus position was larger than 2 mm, the −6 dB focal volume usually matched quite closely in size and shape, as previously discussed for skull B at 270 kHz. Outliers in spatial peak pressure location error were also observed at 1 MHz for skull A in PW mode, with an error $ \triangle z$ of −6 mm for the bowl source, skull B in CW mode where the errors were 8.5 mm with the bowl source and 7.5 mm with the hologram source, and skull D in PW mode with an error −6 mm for the bowl source. High errors for skull B could be explained by its non-uniform thickness. Overall, for 75% of investigated cases the error in peak location was lower than 2 mm. This suggests that in general, the dependency of sound speed on density, determined from equation (1), together with the electron density calibration performed for each CT image, which ensures accurate mapping of the density distribution over the skull, is appropriate for prediction of the acoustic field distribution for these skulls.

### 4.4. Errors across skull samples

A key issue for consideration is the applicability of values of attenuation coefficient obtained from the literature and choice of density to sound speed mapping for simulating ultrasound propagation through the skull. In the context of planning transcranial ultrasound interventions in humans, direct measurement these parameters may not be feasible. This lack of precise, individualised data may increase uncertainty in predictions of the acoustic field within the brain. To examine the effect of these factors here, we combine data for all conditions, to compare the average error in simulations between skulls. Figures 7(d)–(f) present the summary statistics for error in simulated spatial peak pressure, volume of the focal area and axial location of the spatial peak pressure for each skull sample.

As seen in figure 7(d), the most accurate predictions of spatial peak pressure are in the mid-frequency range of 500–750 kHz, where the median error $q_{0.5}(\varepsilon_p)$ ranges from −10% to 4% for all source and emission modes. The higher errors at 270 kHz and 1 MHz may suggest that the linear power law used here does not accurately describe the attenuation in the skulls.

Skull B, which has the highest density, shows a close match between experiment and simulation. The errors for skull A, which also has relatively high density, are also relatively small, with the *ε*_*p*_ less than 12% for frequencies up to 750 kHz. The greatest discrepancy is seen for skull C, which has the most porous structure and relatively low average density. In particular, for this skull, numerical calculations at the lowest frequency underestimate the peak pressure amplitude more than for other skulls, while at the highest frequency, they overestimate it the most.

**Figure 8.**
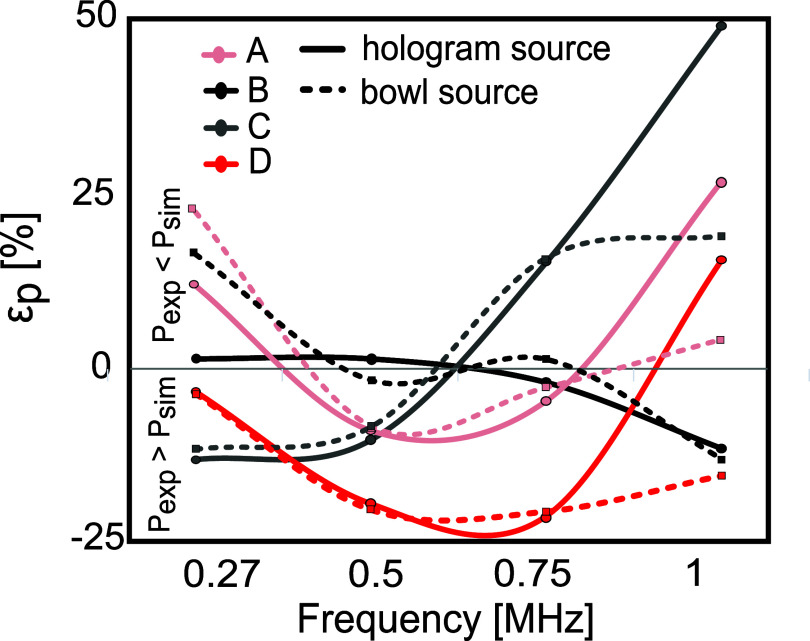
Error in determining spatial peak positive pressure in PW mode, with the hologram and bowl sources for each skull.

The simulated focal pressure amplitude is greatly influenced by the absorption coefficient value defined in the simulations in accordance with Pinton *et al* (2012) and is the same for the entire skull area. In reality, the skull’s attenuation is likely to be non-uniform, and significant deviations from the defined values are possible. This is illustrated by the resulting dependence of errors in spatial peak pressure on frequency which can be seen to vary between skulls. This is shown in figure 8 which presents the difference between the measured and simulated spatial peak pressure for the PW mode, where a positive error indicates that the simulated peak pressure is greater than the measured pressure. As can be seen, in general, measured pressure is lower than simulated at 1 MHz (attenuation is underestimated), and measured pressure is greater than simulated in most cases for the lower frequencies (attenuation is overestimated). These errors do not vary linearly with frequency across skulls suggesting that the frequency dependence of the attenuation coefficient is not linear. The linear power law does result in peak pressure errors below $\pm 25 \%$ for all skulls used here, for all frequencies, except skull C at 1 MHz. This is promising, but still lies outside the estimated 10% uncertainty in hydrophone measurements combined with the hydrophone calibration uncertainty.

The error in the simulated −6 dB volume of the focal zone *ε*_*V*_ as a function of frequency is shown in figure 7(e). It is noticeable that the error increases with frequency, and the simulations in CW mode tend to underestimate the focal volume compared to the measurement. For two of the four skulls, B and D, the focal region was significantly distorted, with the focus split into multiple regions, with increasing distortion as frequency increased. In these cases, small differences in the shape and amplitude of the −6 dB zone between experiment and simulation can lead to greater errors, especially at 1 MHz, where *ε*_*V*_ exceeded 81% for skull B. Meanwhile, for the less aberrated field inside skull C, *ε*_*V*_ was much smaller at −14% for PW mode with the hologram source. The greatest errors in focal position $\triangle z$ and size $(\triangle Lx, \triangle Ly, \triangle Lz)$, occurred at frequencies of 270 kHz and 1 MHz (see figure 7(f) and tables S1–S4), as discussed previously (section 4.3). Therefore, prediction of the position and size of the focus is most accurate in the mid-frequency range.

### 4.5. Bowl source and hologram source

The results of transcranial simulations performed with the optimised uniform pressure bowl source were compared with simulations using the equivalent mass source derived from measured holograms. As shown in figures 5 and 6, pressure profiles and spatial distributions for the hologram source and bowl source are very similar, replicating the amplitude and beam shape well in most cases. Figure 8 illustrates that the disparity between measured and simulated spatial peak pressures in PW mode exhibits comparable behaviour for both hologram and bowl sources. Difference in amplitude and shape is noticeable mainly at 1 MHz, especially for skull D with a distorted field. In most individual cases, the bowl source results in errors similar to that of the hologram, as shown in figure 7. For example, for skulls D and C, *ε*_*p*_ for the hologram and bowl sources were within 3% of each other for frequencies ranging from 270 to 750 kHz in both CW and PW modes (e.g. −13%, −10% and 15% for the hologram source vs −12%, −8% and 16% for the bowl source for skull C in PW mode, see tables S3 and S4).

In almost all cases, the median accuracy of the bowl source across frequencies and skull samples is comparable to that of the hologram source ($q_{0.5}(\varepsilon_p) = -$7% vs −7% for 500 kHz in CW mode, −4% vs −2% for 750 kHz in PW mode), except at the very lowest and highest frequencies in pulsed mode, as shown in figure 7(d). The larger discrepancy at 270 kHz in PW mode ($q_{0.5}(\varepsilon_p) = -$2% vs 7% for hologram and bowl sources respectively) may be due to the effects of the nonuniform pressure distribution on the vibrating surface of the transducer due to Lamb waves, which may play a larger role in the generated field at this frequency.

Additionally, the error in the focus location and the focal volume with the bowl sources generally was not significantly higher than for the hologram source. $q_{0.5}(\varepsilon_V)$, $q_{0.5}(\triangle z)$ were slightly higher for skull D, as shown in figures 7(b) and (c)), which can be explained by the strong distortion of the field. An increase in $q_{0.5}(\triangle z)$ for the bowl source at 500 kHz (figure 7(f)), may be explained by an imperfect bowl source model, as discussed in section 4.1 and shown in figure 3(b). Despite the error in the axial position of the spatial peak pressure, $\triangle z$ across all skulls was slightly higher for the bowl source, but remained under 2 mm in most cases (see figures 7(c) and (f)).

In certain cases, the bowl source model yielded higher accuracy compared to the measured hologram source. This occurred for skulls C and A, with a greater increase at 1 MHz. For example, in the PW mode at 1 MHz, the error *ε*_*p*_ for skull A decreased from 27% with the hologram source to 4% with the bowl source, and for skull C, it decreased from 49% to 19%. For PW at 1 MHz, median error across skull samples was higher for the hologram source ($q_{0.5}\varepsilon_p) = $ 22%) than for the bowl source ($q_{0.5}\varepsilon_p) = -$5%), see figure 7(d). A potential source of uncertainty with a hologram source may arise as the source surface pressure distribution is often not perfectly axially symmetric, so small differences may be introduced if the orientation of the transducer during hologram measurement does not match its orientation in subsequent skull measurements.

**Figure 9.**
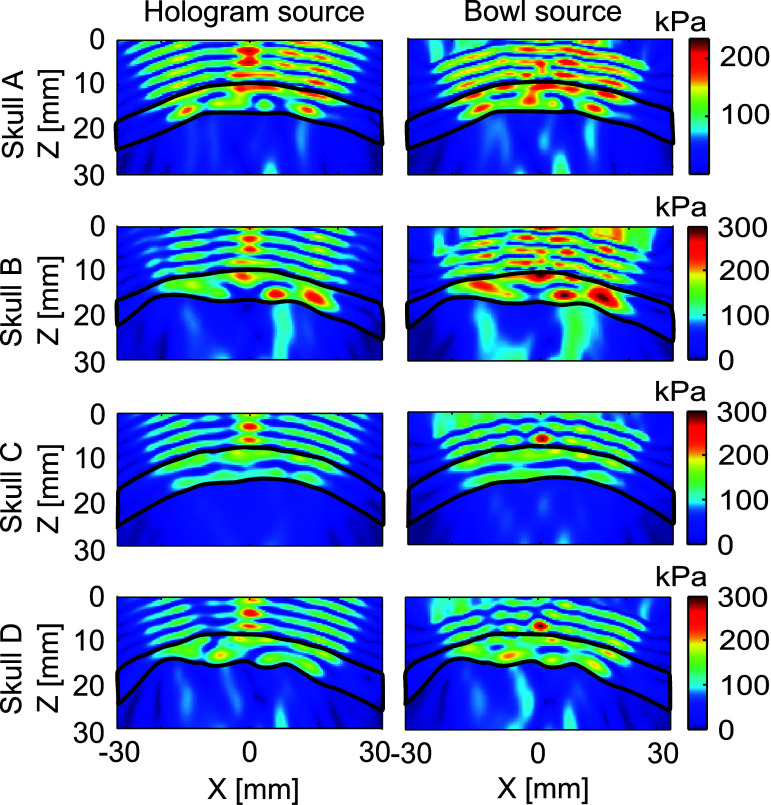
Determination of local pressure maxima within the skull in CW mode at 270 kHz, for the hologram source and the uniform pressure bowl source.

Prediction of pressure within the skull bone itself, and in particular regions of high pressure which may lead to higher temperature rises, is important for safety assessment in transcranial ultrasound therapies. In the context of this study, this is of particular interest when comparing the fields generated by the hologram and bowl sources, where the near field pressures differ. Figure 9 illustrates the distribution of peak positive pressure at 270 kHz in CW mode for a slice through each skull in the *XZ* plane for the hologram and bowl sources. As can be seen in the figures, the pressure distribution within the skull is generally similar for the two sources, and areas of high pressure amplitude appear in similar locations. However, with the hologram source, there is a higher pressure region on the beam axis near the outer surface of the skull in all instances, the amplitude of which is comparable to the focal pressure. This could be associated with the non-uniform pressure distribution on the surface of a real transducer and lead to differences in prediction of temperature rises when using the two models. For the bowl source, some regions of high pressure can within the skull are up to 15% higher in amplitude compared to the hologram source.

### 4.6. Pulse wave and continuous wave signal modes

For applications including thermal ablation and neuromodulation, long pulses are used, where a steady state is reached during the exposure. In this case the total pressure field includes the sum of multiple signal reflections from the transducer surface and the skull.

**Figure 10.**
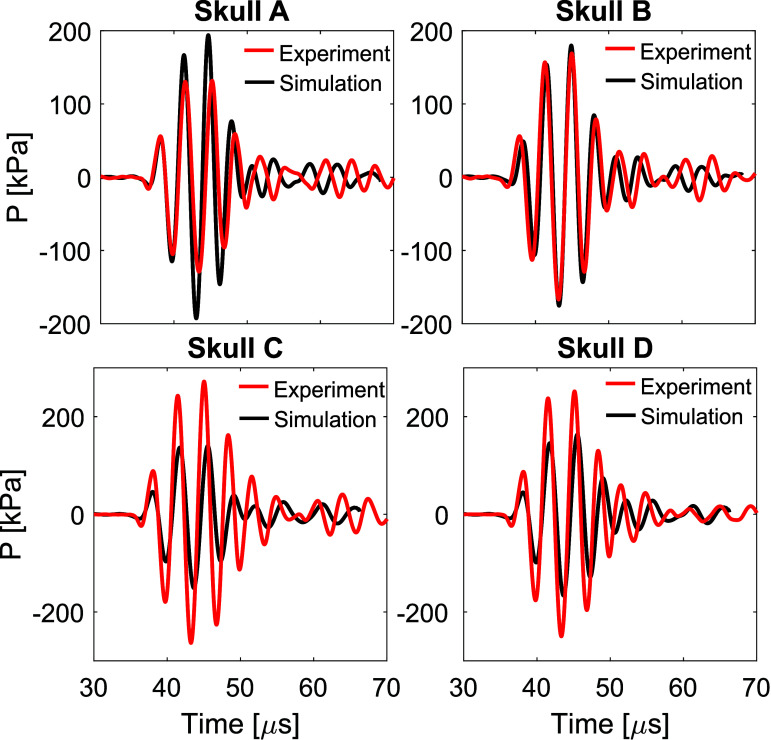
Comparison of measured and simulated focal waveforms at 270 kHz in PW mode at the same position in the field, 55 mm from the transducer.

In this study the short pulse PW mode was included to enable easier comparison of arrival times of the main pulse and reflections in simulations and experiments, to aid in assessment of the suitability of the sound speed mapping. Good agreement in arrival times was typically observed, as shown in figure 10 for all skulls at 270 kHz. Simulations were performed for the hologram source, and comparisons with experiments were made at 55 mm from the transducer surface on axis. As seen from the figures, the amplitude of signals is different in some of the cases due to differences in the aberration between measurements and simulations at this location. Nevertheless, the shape of the main pulse and the arrival times match well. Typically, the error in the spatial peak pressure amplitude is lower with the short-pulse signals than in CW mode ( figures 7(a) and (d)). At lower frequencies, additional errors may arise from interference of longer pulses causing increased reflections between the skull and the transducer. This is consistent with the lower median errors $q_{0.5}(\varepsilon_p)$ in pulsed mode than in quasi-continuous mode (−2% vs −15% for 270 kHz, 22% vs 30% for 1 MHz), although they are comparable in the mid-frequency range. Reflections between the skull and the transducer occur, but arrive later than the main pulse. Near the focus, the waveforms are relatively simple and the reflections are usually small compared to the main pulse, so they do not greatly affect the measured peak pressure amplitude.

**Figure 11.**
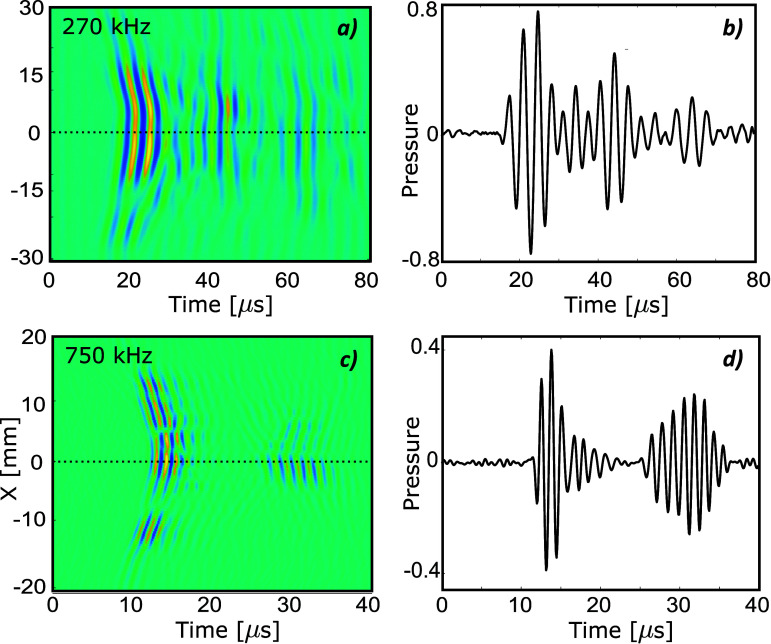
Measured acoustic pressure as a function of time (a), (c) along *X*-axis at *Z* = 20 mm from the transducer surface and (b), (d) at point with coordinates (0, 0, 20) mm for skull B in PW mode at (a), (b) 270 kHz and (c), (d) 750 kHz.

Closer to the transducer, as shown in figure 11 at 20 mm from the transducer surface for 270 and 750 kHz in PW mode, the amplitude of the reflected signals is much larger and can be comparable to the main pulse in some locations. However, the direct signal can usually be separated from subsequent reflections except at 270 kHz, where the direct and reflected signals overlap, because of the long temporal duration of the pulse (figures 11(b) and (d)). This may be one reason for the larger error in spatial peak pressure at 270 kHz for the CW sources compared to the PW sources. The distance separating direct and reflected signals in both cases corresponds to the distance between the transducer and the skull surface suggesting this is the main source of these reflections. In quasi-continuous mode, the long pulse results in overlap of the direct and reflected signals once the field has reached steady state, which can lead to significant (in some cases up to 20%) amplitude fluctuations depending on the measurement window. This leads to higher errors in spatial peak pressure in this case. The source as defined in k-Wave does not include a reflecting surface, and the influence of incident reflections on the source output is not known; therefore, reflections from the transducer are not modelled, which will likely lead to errors in simulation compared to measurement. Including reflections from the source may help to improve agreement between simulation and measurements when long pulses are simulated.

## 5. Discussion

The main challenge in simulating the propagation of ultrasound through the skull is obtaining accurate descriptions of the acoustic medium properties and geometry of the skull. The skull causes aberration and attenuation of the ultrasound by varying amounts depending on the individual skull shape and internal structure, as well as the frequency of ultrasound. The average transmission loss depends linearly on frequency, from −8.3 dB at 270 kHz to −18.9 dB at 1 MHz in average, but variations between individual skull samples can be significant. It is important to note that our study was limited to four skull samples, and four frequencies, with a single source position, so does not capture all possible conditions or cases. The chosen frequency range and normal incidence transducer positioning represent commonly used neuromodulation set ups, enabling the outcomes of this work to be applicable to those studies, especially where k-Plan is used for sonication planning.

In general it is likely that the largest sources of error arise from the mapping of acoustic medium properties and geometry from CT images. Here and in other studies in the literature, the density of the skull at each point is obtained by mapping from CT HUs (Aubry *et al* 2003), in our case using an electron density calibration. The following step of converting density into speed of sound may introduce significant uncertainty (Stanziola *et al* 2023). Several studies demonstrate a linear relationship between density and sound speed over a wide frequency range, but the coefficients of linear regression vary between studies (Connor *et al* 2002, Pichardo *et al* 2010).

However, despite substantial distortion of the field by the skull, the spatial pressure distribution and focal zone are well described by simulations. Experimental errors could occur due to transducer inclination relative to the measurement plane or small misalignments in the skull position in the experimental setup. In most cases, our results demonstrate agreement in spatial distribution of pressure with errors in focal position of 2.7 mm, focal volume within 35% and focal length of 2.3 mm on average, in general with good matching of the overall field shape. The axial focal lengths are approximately 40 mm at 270 kHz, 20 mm at 500 kHz, 12 mm at 750 kHz, and 10 mm at 1 MHz. In comparison, the 2.7 mm error in focal position (or 1.9 mm without outliers at low frequency) is relatively small at low frequencies increasing to approximately 25% at higher frequencies. However, in these cases, when aberration did not cause focal splitting, the position errors were on the order of 1 mm. The size of brain stimulation targets may range from e.g. ${\sim}4\times4\times5$ mm in the deep brain (Martin *et al* 2024) to e.g. ${\sim}16\times7$ to 21 mm in subcortical regions (Robertson *et al* 2017) and larger in the cortex. Many ultrasound neuromodulation studies to date target cortical regions using spherically focusing transducers similar to those used here, with consequently similar focal sizes, which may be similar in size to or larger than the brain region of interest, particularly in the axial direction (Matt *et al* 2024). In this context, this order of error in predicted focal position and dimensions is unlikely to result in missing the target region of interest completely.

For deep brain applications, for example FUS thalamotomy, the ventral intermediate nucleus which has a size of 6–10 mm is targeted (Baek *et al* 2022). The ExAblate Neuro 4000 (InSightec Inc.) system used to deliver this treatment generates a $1.5\times1.5\times3$ mm focus after aberration correction, with positioning accuracy of approximately 1 mm (Leung *et al* 2022). Several sonications may be delivered to produce a necrotic lesion size of approximately $5.6\times14.6\times11$ mm (Harary *et al* 2018). For this application, the focal position errors found here may be unacceptable. However, these treatments are always done under MR guidance to identify and correct the focal position to coincide with the target. In addition, MR thermometry is used to verify the temperature rise to ensure tissue ablation, so accurate prediction of focal pressure amplitude is not used. However, for ultrasonic neuromodulation, other than MR-ARFI, which has been recently demonstrated in humans in this context (Mohammadjavadi *et al* 2024), at present there are no widely available methods for evaluating target engagement, and most studies rely on prediction of *in situ* pressure by simulation. As demonstrated by Robertson *et al* (2017), the spatial distribution of the ultrasound field is only weakly affected by variations in the attenuation coefficient. This suggests that inaccuracies in the specified attenuation coefficient is the main source of error in the simulated spatial peak pressure amplitude in our study, and that improved and individual mapping of the attenuation coefficient has the potential to increase the accuracy of simulations. Consequently, due to large errors of up to 60% in the spatial peak pressure in some cases, it is imperative to reserve a safety margin to mitigate risks when planning transcranial ultrasound studies and interventions using this simulation method. Reducing these errors would also enable more consistent studies, enabling better comparison of results and identification of thresholds for effect. A uniform absorption coefficient was applied across the entire skull volume in this study, which appears to be a major limitation. Previous studies (Leung *et al* 2019) have employed different mappings from HU, which results different attenuation curves: some are based on porosity (Aubry *et al* 2003), while others are based on polynomial relationships with HU fitted from study data (Pinton *et al* 2012, McDannold *et al* 2019), while some use fixed values (Pinton *et al* 2012, Robertson *et al* 2017). In our work, we utilized an attenuation coefficient of 13.3 dB MHz cm^−1^, derived from (Pinton *et al* 2012), which is incorporated in k-Plan. This coefficient, obtained experimentally in the referenced study, was averaged over a limited number of samples at 1 MHz. It does not fully represent the diversity of skull properties across different populations, but falls within the typical attenuation coefficient range at 1 MHz documented in the literature (Fry and Barger 1978, White *et al* 1978, Pichardo *et al* 2010, Marsac *et al* 2017, Webb *et al* 2020). However, this variety in measured values is consistent with variation in errors across skulls seen in this work, supporting the conclusion that these errors arise due to the variation in attenuation coefficient between skulls.

In addition, the dependence of skull attenuation coefficient on frequency is not well established. Some studies have shown a linear correlation between frequency and attenuation coefficient (White *et al* 1978, Connor *et al* 2002, Bossy *et al* 2005), while others have modelled attenuation using power law relationships (Fry and Barger 1978, Webb *et al* 2020). Figure 8 shows that the linear dependence of frequency used is not suitable for describing the most porous skulls but seems to be more suitable for the most dense skulls. Skull D which has the largest size and the lowest average density, has the highest errors in spatial peak pressure in the mid-frequency range, however they are lower compared to other skulls at low and high frequency. The dependence of $ < \varepsilon_p > $ with frequency is not consistent across skulls, indicating that the attenuation in skulls does not vary linearly with frequency and is dependent on the skull microstructure. This is consistent with findings in the literature that bone attenuation depends on the internal structure, i.e. porosity and defects (Bossy *et al* 2005), and is not linearly dependent on frequency (Webb *et al* 2020). Additionally, findings reported in Pichardo *et al* (2010) indicate that the attenuation coefficient shows dependence on the skull density that varied with the frequency. At lower frequencies, it is not always linear (Fry and Barger 1978, Chaffai *et al* 2000). It was noted in Fry and Barger (1978), that the quadratic dependence of scattering loss on frequency is based on Rayleigh scattering theory, which is valid for frequencies low enough so that the diameter of pores inside skull is less than one-third wavelength of ultrasound. At higher frequencies the loss per unit distance will increase as the second power of frequency. This has been demonstrated experimentally by measurement of cancellous bone attenuation coefficient over a wide frequency range for different samples, which shows a power law modification at frequencies around 1 MHz (Chaffai *et al* 2000). It is clear that the determination of the attenuation coefficient for the skull is a subject that requires further research.

Another challenge in mapping the skull geometry and internal structure is the resolution of clinical CT scans (0.47 × 0.47 × 0.5 mm for the images obtained in this study), which is not sufficient to capture the microstructure of the skull. This may present a barrier to obtaining more detailed and individualised estimates of attenuation coefficient based on porosity and microstructure for example, although estimates based on the voxel density may offer some information.

In terms of errors associated with measurements used for comparison, one factor that may introduce errors is skull degassing. Typically, skulls are degassed for 48 h prior to experiments, however, for some samples, a longer time or lower vacuum pressure was required as air bubbles continued to emerge even after this period. The presence of air in the internal structure of the bone could artificially increase the attenuation coefficient in isolated cases. It is essential to consider appropriate skull degassing before performing CT and acoustic experiments, to reduce the possibility of errors. Additionally, there are likely to be differences in the properties of older *ex vivo* skulls which have been stored compared to *in vivo* skull. However, the images from which the density and sound speed were mapped were obtained after the skulls had been soaked and degassed in preparation for measurements. It is also crucial to take into account other sources of experimental errors including errors in positioning and registration of skulls, fluctuations in the source and drive voltage, and hydrophone calibration uncertainty. Plastic holders can slightly shift under the weight of the skull, adding uncertainly in skull position, estimated to be within 1 mm. In Martin and Treeby (2019) it was shown that the stability of hydrophones and source driving conditions, together with careful alignment, enable repeatable measurements, with peak pressures expected to lie within 3% of the mean. The variations attributed to spatial averaging effects, hydrophone positioning, and calibration uncertainties are quoted as 10%.

There are also some limitations to the modelling approach used here, in that a fluid model was used, which does not account for all wave modes. Fluid models of propagation through solid materials have been shown to be accurate for close to normal incidence (White *et al* 2006, Treeby and Saratoon 2015). Gao *et al* (2023) showed that at 500 kHz at normal incidence, the influence of elastic waves in the skull on the overall field pattern is negligible. It has also been shown (Jiang *et al* 2020) that if the incident wave does not exceed the critical angle, shear waves can be neglected. However, including the propagation of elastic waves in the skull can alter the focal position. Calculations of the transcranial focusing by a multi element array transducer at a central frequency of 0.8 MHz resulted in differences in focal position of up to 1 mm when shear waves were modelled compared to a fluid model of skull propagation (Jiang *et al* 2020). In our experiments, the error $\triangle z$ was highest at 270 kHz, especially passing through the most aberrating skull B. In this case the measured spatial peak pressure was located at $z_0 = 38.5$ mm from the transducer, while the calculated *z*_0_ positions for the hologram and bowl sources were 56.5 and 52.5 mm, respectively. This may be attributed to the effect of guided waves propagating along the skull’s surface. As shown by Estrada *et al* (2018), Minonzio *et al* (2018), the first few Lamb wave modes can be observed experimentally, and it has been shown numerically and experimentally, that the first modes have the strongest dispersion in the sub-400 kHz range (Mazzotti *et al* 2021), so their contribution to the field near the skull is likely to decrease with increasing frequency. In addition, in low frequency cases, discrepancies in the near-field structure are evident in both examples shown in figure 6, which could be due in part to the presence of sound speed dispersion in the skull caused by guided acoustic waves (Estrada *et al* 2017, Mazzotti *et al* 2021), or wave mode conversion (Pichardo *et al* 2017, Wang *et al* 2018, Gao *et al* 2023). Thus, neglecting elastic wave modes could be a reason for discrepancies in the spatial peak position, particularly at low frequencies. However, across all frequencies and more significantly so at higher frequencies, the lack of mapping of the skull microstructure still means that scattering and complex wave interactions within the skull are not fully modelled here. These limitations should be considered when interpreting simulation results and planning studies or clinical interventions.

In terms of the influence of the modelled source conditions, fields simulated using uniform pressure bowl and hologram sources achieve similar accuracy in spatial peak pressure, focus position, and volume of the focal zone. At high frequency, the bowl source may even yield more accurate results. The spatial distribution of the pressure field created by the bowl source accurately reproduces the measured field even close to the surface of the skull, and in the case of very distorted fields. The use of a bowl source with optimised geometry parameters is a convenient alternative to a hologram, as it can be defined through simpler, faster measurements, and will have lower memory requirements, reducing the demand on computational resources.

## 6. Summary and conclusion

Simulations of the propagation of ultrasound through *ex vivo* human skull bones predicted on average the absolute values of the spatial peak pressure amplitude to within $15\% \pm 13$%, the focal position to within $2.7 \pm 4.2$ mm, the −6 dB focal volume to within $35\% \pm 42$%, and the -6 dB lateral and axial focal sizes to within $0.3 \pm 0.4$ mm, $0.3 \pm 0.3$ mm, and $ 2.3 \pm 2.3$ mm while also reproducing the complex field structure within the skull cavity. Defining the source as a bowl with uniform distribution of amplitude and pressure phase on the surface achieves the same order of calculation accuracy as hologram sources, provided that the radii of curvature and apertures are corrected at each frequency. The bowl source model proves to be a practical alternative to holograms for intracranial field prediction for therapy planning. The results of this study suggest that the chosen uniform linear attenuation coefficient mapping was the largest source of errors for the skull samples employed in this study. Skull specific mapping of skull attenuation will be the subject of further work.

In summary, the results of this work demonstrate the feasibility of simulation of the propagation of ultrasound through the skull using k-Wave with CT derived acoustic medium property maps for prediction of focal position, size and overall field distribution. The possibility of considerable errors in pressure amplitude should be taken into account when planning therapeutic studies or interventions in the human brain using focused ultrasound, and an appropriate safety margin should be used.

## Data Availability

The data that support the findings of this study will be openly available following an embargo at the following URL/DOI: https://doi.org/10.5522/04/28025240.

## References

[pmbada19dbib1] Alexander S L, Rafaels K, Gunnarsson C A, Weerasooriya T (2019). Structural analysis of the frontal and parietal bones of the human skull. J. Mech. Behav. Biomed. Mater..

[pmbada19dbib2] Almquist S, Parker D L, Christensen D A (2016). Rapid full-wave phase aberration correction method for transcranial high-intensity focused ultrasound therapies. J. Ther. Ultrasound.

[pmbada19dbib3] Angla C, Larrat B, Gennisson J, Chatillon S (2023). Transcranial ultrasound simulations: a review. Med. Phys..

[pmbada19dbib4] Atkinson-Clement C, Howett D, Alkhawashki M, Ross J, Slater B, Gatica M, Zhang C, Petkov C I, Kaiser M (2023). Extended temporal dynamics of transcranial ultrasound stimulation in the primate brain. SSRN 4653169.

[pmbada19dbib5] Aubry J-F (2022). Benchmark problems for transcranial ultrasound simulation: intercomparison of compressional wave models. J. Acoust. Soc. Am..

[pmbada19dbib6] Aubry J-F, Tanter M, Pernot M, Thomas J-L, Fink M (2003). Experimental demonstration of noninvasive transskull adaptive focusing based on prior computed tomography scans. J. Acoust. Soc. Am..

[pmbada19dbib7] Baek H, Lockwood D, Mason E J, Obusez E, Poturalski M, Rammo R, Nagel S J, Jones S E (2022). Clinical intervention using focused ultrasound (FUS) stimulation of the brain in diverse neurological disorders. Front. Neurol..

[pmbada19dbib8] Bancel T, Houdouin A, Annic P, Rachmilevitch I, Shapira Y, Tanter M, Aubry J-F (2021). Comparison between ray-tracing and full-wave simulation for transcranial ultrasound focusing on a clinical system using the transfer matrix formalism. IEEE Trans. Ultrason. Ferroelectr. Freq. Control.

[pmbada19dbib9] Bao S, Kim H, Shettigar N B, Li Y, Lei Y (2024). Personalized depth-specific neuromodulation of the human primary motor cortex via ultrasound. J. Physiol..

[pmbada19dbib10] Bossy E, Padilla F, Peyrin F, Laugier P (2005). Three-dimensional simulation of ultrasound propagation through trabecular bone structures measured by synchrotron microtomography. Phys. Med. Biol..

[pmbada19dbib11] Bouchoux G, Bader K B, Korfhagen J J, Raymond J L, Shivashankar R, Abruzzo T A, Holland C K (2012). Experimental validation of a finite-difference model for the prediction of transcranial ultrasound fields based on CT images. Phys. Med. Biol..

[pmbada19dbib12] Chaffai S, Padilla F, Berger G, Laugier P (2000). In vitro measurement of the frequency-dependent attenuation in cancellous bone between 0.2 and 2 MHz. J. Acoust. Soc. Am..

[pmbada19dbib13] Chaplin V, Phipps M A, Caskey C F (2018). A random phased-array for MR-guided transcranial ultrasound neuromodulation in non-human primates. Phys. Med. Biol..

[pmbada19dbib14] Clement G T, Hynynen K (2002). A non-invasive method for focusing ultrasound through the human skull. Phys. Med. Biol..

[pmbada19dbib15] Clement G T, White P J, Hynynen K (2004). Enhanced ultrasound transmission through the human skull using shear mode conversion. J. Acoust. Soc. Am..

[pmbada19dbib16] Coluccia D, Fandino J, Schwyzer L, O’Gorman R, Remonda L, Anon J, Martin E, Werner B (2014). First noninvasive thermal ablation of a brain tumor with MR-guided focusedultrasound. J. Ther. Ultrasound.

[pmbada19dbib17] Connor C W, Clement G T, Hynynen K (2002). A unified model for the speed of sound in cranial bone based on genetic algorithm optimization. Phys. Med. Biol..

[pmbada19dbib18] Deffieux T, Konofagou E E (2010). Numerical study of a simple transcranial focused ultrasound system applied to blood-brain barrier opening. IEEE Trans. Ultrason. Ferroelectr. Freq. Control.

[pmbada19dbib19] Ding X, Wang Y, Zhang Q, Zhou W, Wang P, Luo M, Jian X (2015). Modulation of transcranial focusing thermal deposition in nonlinear HIFU brain surgery by numerical simulation. Phys. Med. Biol..

[pmbada19dbib20] Douglas Mast T (2000). Empirical relationships between acoustic parameters in human soft tissues. Acoust. Res. Lett. Online.

[pmbada19dbib21] Estrada H, Gottschalk S, Reiss M, Neuschmelting V, Rebling J, Goldbrunner R, Razansky D (2018). Looking at the skull in a new light: Rayleigh-lamb waves in cranial bone.

[pmbada19dbib22] Estrada H, Rebling J, Razansky D (2017). Prediction and near-field observation of skull-guided acoustic waves. Phys. Med. Biol..

[pmbada19dbib23] Fedorov A (2012). 3D slicer as an image computing platform for the quantitative imaging network. Magn. Reson. Imaging.

[pmbada19dbib24] Fomenko A, Neudorfer C, Dallapiazza R F, Kalia S K, Lozano A M (2018). Low-intensity ultrasound neuromodulation: an overview of mechanisms and emerging human applications. Brain Stimul..

[pmbada19dbib25] Franzini A, Moosa S, Prada F, Jeffrey Elias W (2020). Ultrasound ablation in neurosurgery: current clinical applications and future perspectives. Neurosurgery.

[pmbada19dbib26] Fry F J, Barger J E (1978). Acoustical properties of the human skull. J. Acoust. Soc. Am..

[pmbada19dbib27] Gao Y, Werner B, Lauber B, Chen Y, Colacicco G, Razansky D, Estrada H (2023). Influence of shear waves on transcranial ultrasound propagation in cortical brain regions. https://arxiv.org/abs/2309.12838.

[pmbada19dbib28] Harary M, Essayed W I, Valdes P A, McDannold N, Rees Cosgrove G (2018). Volumetric analysis of magnetic resonance–guided focused ultrasound thalamotomy lesions. Neurosurg. Focus.

[pmbada19dbib29] Jiang C, Li D, Xu F, Li Y, Liu C, Ta D (2020). Numerical evaluation of the influence of skull heterogeneity on transcranial ultrasonic focusing. Front. Neurosci..

[pmbada19dbib30] Jones R M, Hynynen K (2015). Comparison of analytical and numerical approaches for CT-based aberration correction in transcranial passive acoustic imaging. Phys. Med. Biol..

[pmbada19dbib31] Kaloev A Z, Nikolaev D A, Khokhlova V A, Tsysar S A, Sapozhnikov O A (2022). Spatial correction of an acoustic hologram for reconstructing surface vibrations of an axially symmetric ultrasound transducer. Acoust. Phys..

[pmbada19dbib32] Kamimura H A S, Conti A, Toschi N, Konofagou E E (2020). Ultrasound neuromodulation: mechanisms and the potential of multimodal stimulation for neuronal function assessment. Front. Phys..

[pmbada19dbib33] Krokhmal A, Simcock I, Treeby B, Martin E (2024). A Comparative Study of Experimental and Simulated Ultrasound Beam Propagation Through Cranial Bones Repository name: DRUM: Data-repository for biomedical ultrasound metrology, UCL research data repository.

[pmbada19dbib34] Leduc N, Okita K, Sugiyama K, Takagi S, Matsumoto Y (2012). Focus control in hifu therapy assisted by time-reversal simulation with an iterative procedure for hot spot elimination. J. Biomech. Sci. Eng..

[pmbada19dbib35] Leung S A, Moore D, Gilbo Y, Snell J, Webb T D, Meyer C H, Wilson Miller G, Ghanouni P, Butts Pauly K (2022). Comparison between MR and CT imaging used to correct for skull-induced phase aberrations during transcranial focused ultrasound. Sci. Rep..

[pmbada19dbib36] Leung S A, Webb T D, Bitton R R, Ghanouni P, Butts Pauly K (2019). A rapid beam simulation framework for transcranial focused ultrasound. Sci. Rep..

[pmbada19dbib37] Maimbourg G, Houdouin A, Deffieux T, Tanter M, Aubry J-F (2018). 3D-printed adaptive acoustic lens as a disruptive technology for transcranial ultrasound therapy using single-element transducers. Phys. Med. Biol..

[pmbada19dbib38] Marquet F, Pernot M, Aubry J-F, Montaldo G, Marsac L, Tanter M, Fink M (2009). Non-invasive transcranial ultrasound therapy based on a 3D CT scan: protocol validation and in vitro results. Phys. Med. Biol..

[pmbada19dbib39] Marsac L, Chauvet D, Greca R L, Boch A-L, Chaumoitre K, Tanter M, Aubry J-F (2017). Ex vivo optimisation of a heterogeneous speed of sound model of the human skull for non-invasive transcranial focused ultrasound at 1 MHz. Int. J. Hyperth..

[pmbada19dbib40] Martin E (2024). Ultrasound system for precise neuromodulation of human deep brain circuits.

[pmbada19dbib41] Martin E, Jaros J, Treeby B E (2019). Experimental validation of k-wave: nonlinear wave propagation in layered, absorbing fluid media. IEEE Trans. Ultrason. Ferroelectr. Freq. Control.

[pmbada19dbib42] Martin E, Treeby B (2019). Investigation of the repeatability and reproducibility of hydrophone measurements of medical ultrasound fields. J. Acoust. Soc. Am..

[pmbada19dbib43] Matt E, Radjenovic S, Mitterwallner M, Beisteiner R (2024). Current state of clinical ultrasound neuromodulation. Front. Neurosci..

[pmbada19dbib44] Mazzotti M, Sugino C, Kohtanen E, Erturk A, Ruzzene M (2021). Experimental identification of high order lamb waves and estimation of the mechanical properties of a dry human skull. Ultrasonics.

[pmbada19dbib45] McDannold N, White P J, Cosgrove R (2019). Elementwise approach for simulating transcranial MRI-guided focused ultrasound thermal ablation. Phys. Rev. Res..

[pmbada19dbib46] Minonzio J-G, Bochud N, Vallet Q, Bala Y, Ramiandrisoa D, Follet H‘ene, Mitton D, Laugier P (2018). Bone cortical thickness and porosity assessment using ultrasound guided waves: an ex vivo validation study. Bone.

[pmbada19dbib47] Mohammadjavadi M, Ash R T, Glover G H, Butts Pauly K (2024). Optimization of MR-ARFI for human transcranial focused ultrasound. bioRxiv Preprint.

[pmbada19dbib48] Mueller J K, Ai L, Bansal P, Legon W (2017). Numerical evaluation of the skull for human neuromodulation with transcranial focused ultrasound. J. Neural Eng..

[pmbada19dbib49] O’Neil H T (1949). Theory of focusing radiators. J. Acoust. Soc. Am..

[pmbada19dbib50] Pasquinelli C, Hanson L G, Siebner H R, Lee H J, Thielscher A (2019). Safety of transcranial focused ultrasound stimulation: a systematic review of the state of knowledge from both human and animal studies. Brain Stimul..

[pmbada19dbib51] Pichardo S, Moreno-Hernández C, Andrew Drainville R, Sin V, Curiel L, Hynynen K (2017). A viscoelastic model for the prediction of transcranial ultrasound propagation: application for the estimation of shear acoustic properties in the human skull. Phys. Med. Biol..

[pmbada19dbib52] Pichardo S, Sin V W, Hynynen K (2010). Multi-frequency characterization of the speed of sound and attenuation coefficient for longitudinal transmission of freshly excised human skulls. Phys. Med. Biol..

[pmbada19dbib53] Pinton G, Aubry J-F, Bossy E, Muller M, Pernot M, Tanter M (2012). Attenuation, scattering and absorption of ultrasound in the skull bone. Med. Phys..

[pmbada19dbib54] Reinhard M, Hetzel A, Krüger S, Kretzer S, Talazko J, Ziyeh S, Weber J, Els T (2006). Blood-brain barrier disruption by low-frequency ultrasound. Stroke.

[pmbada19dbib55] Robertson J L B, Cox B T, Jaros J, Treeby B E (2017). Accurate simulation of transcranial ultrasound propagation for ultrasonic neuromodulation and stimulation. J. Acoust. Soc. Am..

[pmbada19dbib56] Robertson J, Martin E, Cox B, Treeby B E (2017). Sensitivity of simulated transcranial ultrasound fields to acoustic medium property maps. Phys. Med. Biol..

[pmbada19dbib57] Rosnitskiy P B, Yuldashev P V, Sapozhnikov O A, Gavrilov L R, Khokhlova V A (2019). Simulation of nonlinear trans-skull focusing and formation of shocks in brain using a fully populated ultrasound array with aberration correction. J. Acoust. Soc. Am..

[pmbada19dbib58] Salahshoor H, Shapiro M G, Ortiz M (2020). Transcranial focused ultrasound generates skull-conducted shear waves: computational model and implications for neuromodulation. Appl. Phys. Lett..

[pmbada19dbib59] Sapozhnikov O A, Ponomarev A E, Smagin M A (2006). Transient acoustic holography for reconstructing the particle velocity of the surface of an acoustic transducer. Acoust. Phys..

[pmbada19dbib60] Sapozhnikov O A, Tsysar S A, Khokhlova V A, Kreider W (2015). Acoustic holography as a metrological tool for characterizing medical ultrasound sources and fields. J. Acoust. Soc. Am..

[pmbada19dbib61] Schwartz M L, Yeung R, Huang Y, Lipsman N, Krishna V, Jain J D, Chapman M G, Lozano A M, Hynynen K (2018). Skull bone marrow injury caused by mr-guided focused ultrasound for cerebral functional procedures. J. Neurosurg..

[pmbada19dbib62] Sigona M K, Manuel T J, Anthony Phipps M, Banaie Boroujeni K, Louie Treuting R, Womelsdorf T, Caskey C F (2023). Generating patient-specific acoustic simulations for transcranial focused ultrasound procedures based on optical tracking information. IEEE Open J. Ultrason. Ferroelectr. Freq. Control.

[pmbada19dbib63] Stanziola A, Pineda-Pardo J A, Treeby B (2023). Transcranial ultrasound simulation with uncertainty estimation. JASA Express Lett..

[pmbada19dbib64] Stern J M (2021). Safety of focused ultrasound neuromodulation in humans with temporal lobe epilepsy. Brain Stimul..

[pmbada19dbib65] Sukovich J R (2018). *In vivo* histotripsy brain treatment. J. Neurosurg..

[pmbada19dbib66] Treeby B E, Cox B T (2010). k-Wave: MATLAB toolbox for the simulation and reconstruction of photoacoustic wave fields. J. Biomed. Opt..

[pmbada19dbib67] Treeby B E, Saratoon T (2015). The contribution of shear wave absorption to ultrasound heating in bones: coupled elastic and thermal modeling.

[pmbada19dbib68] Treeby B, Lucka F, Martin E, Cox B T (2018). Equivalent-source acoustic holography for projecting measured ultrasound fields through complex media. IEEE Trans. Ultrason. Ferroelectr. Freq. Control.

[pmbada19dbib69] Wang X-D, Lin W-J, Su C, Wang X-M (2018). Influence of mode conversions in the skull on transcranial focused ultrasound and temperature fields utilizing the wave field separation method: a numerical study. Chin. Phys. B.

[pmbada19dbib70] Webb T D, Leung S A, Ghanouni P, Dahl J J, Pelc N J, Butts Pauly K (2020). Acoustic attenuation: multifrequency measurement and relationship to ct and mr imaging. IEEE Trans. Ultrason. Ferroelectr. Freq. Control.

[pmbada19dbib71] White D N, Curry G R, Stevenson R J (1978). The acoustic characteristics of the skull. Ultrasound Med. Biol..

[pmbada19dbib72] White P J, Clement G T, Hynynen K (2006). Longitudinal and shear mode ultrasound propagation in human skull bone. Ultrasound Med. Biol..

[pmbada19dbib73] Yasuda J, Yoshikawa H, Tanaka H (2019). Phase aberration correction for focused ultrasound transmission by refraction compensation. Jpn. J. Appl. Phys..

[pmbada19dbib74] Yoon K, Lee W, Croce P, Cammalleri A, Yoo S-S (2018). Multi-resolution simulation of focused ultrasound propagation through ovine skull from a single-element transducer. Phys. Med. Biol..

[pmbada19dbib75] Young Park T, Joo Pahk K, Kim H (2019). Method to optimize the placement of a single-element transducer for transcranial focused ultrasound. Comput. Methods Programs Biomed..

